# Forest–river interfaces shape lobomycosis risk in the Amazon Basin

**DOI:** 10.1371/journal.pntd.0014502

**Published:** 2026-07-06

**Authors:** Franciely G. Gonçalves, Roberto C. Ilacqua, Luiza A. Vianna, Allan Sbardelott, Vânia L. Q. Barros, Rosineide F. Bispo, Yally A. S. Sbardelott, Marcus M. Teixeira, Gabriel Z. Laporta

**Affiliations:** 1 Graduate Program in Health Sciences, FMABC University Center, Santo André, São Paulo, Brazil; 2 Center for Health Sciences and Sports, Federal University of Acre, Rio Branco, Acre, Brazil; 3 Acre State Dermatology Service, Rio Branco, Acre, Brazil; 4 Department of Tropical Medicine, Faculty of Medicine, University of Brasília, Brasília, Distrito Federal, Brazil; 5 Consultacre, Rio Branco, Acre, Brazil; 6 Institute for Infectious Disease Translational Research, University of South Carolina, Columbia, South Carolina, United States of America; FIOCRUZ: Fundacao Oswaldo Cruz, BRAZIL

## Abstract

**Background:**

Lobomycosis is a chronic implantation mycosis caused by the uncultivable fungus *Paracoccidioides lobogeorgii*. The disease occurs predominantly in the Amazon Basin and has traditionally been associated with traumatic inoculation during exposure to forest environments. However, many patients live, work, or travel in riverine landscapes, suggesting that environmental determinants beyond forest exposure may influence the spatial occurrence of disease.

**Methodology/Principal findings:**

We conducted a spatial case–background study including 192 confirmed lobomycosis cases and two environmental background locations per case. Using annual land use and land cover data aligned with the estimated year of infection, we quantified the composition and configuration of forests and water bodies at two spatial scales: 3 km^2^ and 10 km^2^. Generalized additive models were used to assess associations between environmental variables and lobomycosis occurrence while accounting for spatial structure. Models incorporating landscape configuration outperformed those based on landscape composition alone. Water-body dominance, measured by the largest patch index, and proximity to rivers were associated with lobomycosis occurrence, supporting a role for forest–river interfaces in the spatial distribution of disease.

**Conclusions/Significance:**

These findings suggest that lobomycosis occurrence in the Amazon is shaped not only by forest cover but also by the spatial configuration of forest and aquatic environments. Forest–river interfaces may represent ecological and human activity zones where environmental suitability and exposure opportunities overlap. These results provide new insights into the environmental ecology of lobomycosis and may help guide surveillance and prevention strategies in riverine Amazonian landscapes.

## Author summary

Lobomycosis is a neglected fungal disease that causes chronic skin lesions and mainly affects people living in tropical regions of the Americas, especially in the Amazon. The disease has usually been linked to contact with deep forest environments, but many patients live or work near rivers. In this study, we investigated whether the spatial occurrence of lobomycosis is related not only to forest cover, but also to the way forests and water bodies are arranged in the landscape. We analyzed 192 confirmed cases and compared their surrounding environments with randomly selected background locations from the same municipalities. We found that cases were more strongly associated with landscapes where forest and water bodies form connected riverine environments than with forest cover alone. These findings suggest that forest–river interfaces may be important areas for exposure, possibly because they combine suitable environmental conditions with daily activities such as fishing, farming, transportation, and movement through riparian vegetation. This information may help guide surveillance and prevention in remote Amazonian communities.

## Introduction

Lobomycosis, also known as Jorge Lobo’s disease, is a chronic implantation mycosis caused by the uncultivable fungus *Paracoccidioides lobogeorgii*. The disease occurs mainly in tropical regions of the Americas, with reports from South America and Central America, including the Amazon Basin, Panama, and French Guiana [1–7]. Clinically, lobomycosis is characterized by slowly progressive cutaneous and subcutaneous lesions that may persist for decades, often causing disfigurement and functional impairment [8–11]. Despite decades of research, treatment options remain limited, and the disease continues to be considered one of the most neglected fungal infections affecting tropical populations [3,4,12].

Epidemiological descriptions have traditionally portrayed lobomycosis as a disease associated with forest environments. Cases have historically been reported among Indigenous populations, rubber tappers, forest extractivists, and other workers whose livelihoods depend on activities within dense tropical forests [2,13–17]. This pattern has reinforced the long-standing view that infection risk is closely linked to prolonged exposure to primary forest ecosystems.

Current hypotheses regarding transmission propose that infection occurs through traumatic inoculation of fungal cells into the skin. Minor injuries caused by plant material, such as thorns, branches, or splinters, are frequently described as initiating events in lesion development [15,18]. This mechanism has supported the assumption that the environmental reservoir of the pathogen is associated with forest vegetation and that infection risk increases with direct contact with forest substrates. More recently, animal-associated exposures have also been proposed as possible routes of inoculation, including a reported pediatric case with tick exposure, raising the hypothesis that arthropod-associated trauma, or potentially vector-associated mechanisms, may contribute to transmission in some settings [10].

However, several observations challenge the notion that transmission is restricted to forest environments. A substantial proportion of reported cases occur in individuals whose primary occupations are not directly related to forest extraction. Fishermen and riverine populations represent recurring groups in case series from the Amazon region [2,3], and similar patterns have been reported in northern South America, including Colombia and Bolivia [1,17]. These observations suggest that environmental exposures beyond direct contact with forest vegetation may contribute to disease occurrence.

Spatial observations provide additional clues. Georeferenced reports of lobomycosis frequently occur near major river systems across the Amazon Basin. Case clusters have been described in regions associated with large river corridors, including the Tapajós River in Brazil and the Orinoco River in northern South America [2,14,16]. Rivers structure human mobility, settlement patterns, and subsistence activities throughout the region. However, the ecological relationship between lobomycosis occurrence and river–forest landscapes has not been formally investigated using spatial epidemiological approaches. Landscape metrics describing forest and water composition and configuration offer a framework to quantify how environmental structure may shape disease occurrence [19].

Here, we test the hypothesis that the spatial occurrence of lobomycosis is shaped not only by the presence of forest environments but also by the spatial relationship between forest landscapes and river systems. We conducted a spatial case–background analysis including 192 confirmed cases of lobomycosis in the Amazon region and two environmental background locations for each case. Using annual remotely sensed land use and land cover data, we evaluated forest and water composition and configuration at multiple spatial scales while adjusting for relevant environmental and demographic covariates. By linking landscape metrics to the estimated year of infection, this analysis incorporated temporal variation in land cover across the study period. This approach provides a quantitative assessment of how landscape structure and river proximity are associated with the observed spatial occurrence of lobomycosis.

## Methods

### Ethics statement

This study was conducted in accordance with the ethical standards for research involving human participants. The study protocol was approved by the Ethics Committee of the Acre State Hospital Foundation (FUNDHACRE) (CAAE 82130824.4.0000.5009; approval number 7.010.534). Written informed consent was obtained from all participants for the use of their data in this research.

### Study design and study area

We conducted a spatial case–background study to investigate environmental determinants of lobomycosis occurrence in the Amazon Basin. The analysis included georeferenced cases reported from municipalities across the Brazilian Amazon. For each case, two environmental background locations were randomly selected within the same municipality, resulting in a 1:2 case–background design. Background locations were restricted to the same municipality to ensure comparable environmental contexts but were not individually matched in the statistical models. The study evaluated whether landscape composition, landscape configuration, and proximity to river systems were associated with the spatial occurrence of lobomycosis.

### Georeferencing of cases

Locations of confirmed lobomycosis cases were georeferenced using a multi-step approach. First, available address information from clinical records was standardized and geocoded using municipal address databases. When precise coordinates were unavailable, locations were refined using visual inspection in Google Earth based on locality descriptions, landmarks, or known settlements [20].

To improve spatial accuracy, additional information was obtained through active case investigation, including contact with local health services and field verification when possible. In some instances, coordinates were directly recorded using handheld global positioning system devices during field visits [21].

This approach allowed geographic coordinates to be assigned to each case location with the highest spatial precision achievable given the available information. During clinical anamnesis, patients were asked about the onset of their lesions, including the approximate year in which the lesion was first noticed and the place of residence at that time [10,11,22]. The probable year of infection was then inferred from the reported onset of the lesion, assuming that infection and the appearance of clinically detectable lesions occur within the same or closely related time period. The reported place of residence at the time of lesion onset was used as the primary spatial reference for georeferencing, under the assumption that this location represents the most plausible proxy for the site of infection.

### Selection of background locations

Background locations were sampled within the same municipality as each case and assigned the same temporal reference year used for the corresponding case. For each georeferenced case, two background locations were randomly selected within the same municipality to ensure comparable environmental contexts. Background locations were constrained to be at least 2 km from the corresponding case and at least 2 km from municipal boundaries to prevent buffer overlap and minimize edge effects during landscape metric extraction [23]. This procedure ensured that background locations represented comparable landscapes while avoiding spatial dependence between case and background buffers.

Background locations were defined as environmental pseudo-absence points sampled within the same geographic domain as cases, rather than locations of confirmed disease-free individuals. This case–background design is widely used in spatial epidemiology when true absence data are unavailable, allowing the characterization of environmental contrasts between observed case locations and the underlying landscape [23–25].

### Land use and land cover data

Environmental variables were derived from land use and land cover datasets provided by the MapBiomas project [26]. For each case, the most probable year of infection was estimated as described above, based on clinical history and the reported onset of lesions [10,11,22]. The corresponding annual land cover map was selected to represent landscape conditions in the estimated year of infection, ensuring temporal alignment between environmental variables and the inferred timing of exposure. Land cover data were extracted for the municipality and year corresponding to each case.

When the estimated year of infection preceded 1985, the earliest available dataset (1985) was used as a proxy for baseline landscape conditions. Landscape metrics for both case and background locations were derived from the same temporal reference, ensuring consistency in the characterization of environmental conditions.

Circular buffers defined by areas of 3-km^2^ and 10-km^2^ (equivalent to radii of approximately 1 km and 1.8 km) were generated around each case and background location [21]. These spatial extents were selected to capture environmental conditions at two complementary scales: the immediate surroundings of the georeferenced location and the broader landscape context potentially associated with human activities such as fishing, forest use, and riverine mobility in Amazonian environments [13,17]. Landscape metrics were then extracted within each buffer.

### Landscape metrics

Landscape metrics were used to quantify both landscape composition and configuration within each buffer. Landscape composition was measured using the percentage of landscape (PLAND) occupied by two land cover classes: primary forest and water bodies [21,27].

Landscape configuration was quantified using the largest patch index (LPI), which represents the percentage of the landscape occupied by the largest contiguous patch of a given land cover class. LPI was calculated separately for primary forest and water bodies to capture the spatial dominance of these landscape features [27].

These metrics allowed the assessment of whether lobomycosis occurrence was associated with the amount of forest and water present in the landscape (composition) or with their spatial structure and dominance (configuration) [13].

### Environmental covariates

Additional environmental variables were included as potential confounders. Elevation was extracted from the Shuttle Radar Topography Mission digital elevation model [28]. Population density was obtained from the WorldPop global population dataset [29].

Distance to the nearest river was calculated using hydrographic data from the Brazilian National Water Agency [30]. For each case and background location, the Euclidean distance to the closest mapped river segment was computed [31].

### Statistical modelling

Associations between environmental variables and lobomycosis occurrence were evaluated using generalized additive models (GAMs) implemented in R v. 4.5.1 with the mgcv package [32]. Models were fitted assuming a binomial error distribution with a logit link, with case versus background status as the response variable. GAMs allow flexible modeling of nonlinear relationships between predictors and the response variable. To account for spatial structure, geographic coordinates were included as a two-dimensional smooth term using thin plate regression splines.

Separate models were constructed to compare landscape composition and landscape configuration metrics. Composition models included PLAND metrics for forest and water, whereas configuration models included LPI for the same land cover classes [33]. All models were adjusted for distance to river, elevation, and population density.

Potential collinearity among covariates was assessed using variance inflation factors calculated from equivalent parametric model structures including the same predictors but excluding the spatial smooth term.

Model selection was based on the corrected Akaike Information Criterion (AICc), with lower values indicating better model fit [34]. Candidate models representing different landscape metrics and spatial scales (3-km^2^ and 10-km^2^ buffers) were compared using ΔAICc and Akaike weights.

For the best-supported models, odds ratios (OR) and 95% confidence intervals were derived from the estimated coefficients to quantify the magnitude and direction of associations between environmental variables and lobomycosis occurrence [35].

Spatial autocorrelation in model residuals was evaluated using Moran’s I statistics. A row-standardized inverse-distance spatial weights matrix was constructed from pairwise Euclidean distances among observation coordinates and used to test whether residual spatial structure remained after accounting for environmental predictors. Non-significant Moran’s I values were interpreted as evidence that residual spatial autocorrelation had been adequately addressed by the models [36].

As a sensitivity analysis, we evaluated the robustness of model estimates to temporal heterogeneity in case occurrence. Observations were stratified according to the probable year of infection into two periods (pre-2000 and post-2000), and the best-supported models were refitted separately within each subset. This approach allowed us to assess whether the direction and magnitude of associations between environmental variables and lobomycosis occurrence were consistent across broad temporal periods.

### Spatial analysis workflow

All spatial analyses were conducted in projected planar coordinates. Geographic coordinates were transformed to the SIRGAS 2000 datum using the Universal Transverse Mercator projection appropriate for the study region [37]. Buffers, distance calculations, and landscape metrics were computed in projected coordinate space.

### Data availability

The analytical datasets underlying the findings of this study, including georeferenced coordinates representing the probable location of infection, derived environmental variables, and the full native R analysis script, are available in Zenodo [38].

## Results

A total of 192 georeferenced cases of lobomycosis were included in the analysis (Table 1). Most cases occurred in men (84%) and individuals aged 40–59 years (47%). Most probable locations of infection were reported in Acre, Brazil (64%), with the highest proportion of estimated infections occurring between 1980 and 1999 (46%).

**Table 1:** Demographic, occupational, and epidemiological characteristics of georeferenced lobomycosis cases included in the analysis (n = 192).

Variable	Category	n	%
Sex	Male	161	84
	Female	31	16
Age group (years)	<40	21	11
	40–59	90	47
	≥60	81	42
Occupation	Forest-related activities	75	39
	River-related activities	3	2
	Agriculture and farming	67	35
	Domestic or household activities	21	11
	Urban labor or service occupations	14	7
	Not working	8	4
	Unknown	4	2
Probable location of infection	Acre, Brazil	123	64
	Amazonas, Brazil	49	26
	Rondônia, Brazil	10	5
	Pará, Brazil	1	0.5
	Bolivia (Brazilian border)	8	4
	Peru	1	0.5
Probable year of infection	≤1979	52	27
	1980–1999	89	46
	2000–2019	46	24
	≥2020	5	3

**Fig 1:**
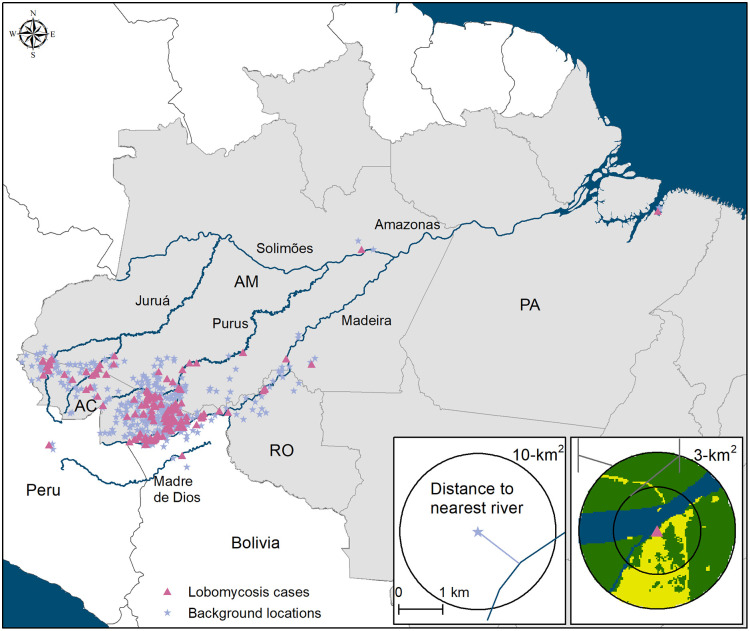
Study design and spatial variables used in the analysis. For each confirmed lobomycosis case, two environmental background locations were randomly selected within the same municipality. The figure illustrates the spatial distribution of cases and background locations, major river systems, the calculation of distance to the nearest river, and the circular buffers of 3-km² and 10-km² used to derive landscape metrics. The inset shows the land cover classification used to quantify landscape metrics: primary forest in dark green; secondary forest, bare soil, and urbanized areas in yellow; and surface water bodies in blue. South American country boundaries and ocean polygons were obtained from Natural Earth (Admin 0 – Countries: https://www.naturalearthdata.com/downloads/10m-cultural-vectors/10m-admin-0-countries/; Ocean: https://www.naturalearthdata.com/downloads/10m-physical-vectors/10m-ocean/), Brazilian administrative boundaries were obtained from the Brazilian Institute of Geography and Statistics (https://www.ibge.gov.br/geociencias/organizacao-do-territorio/malhas-territoriais.html), hydrographic layers from the Brazilian National Water Agency (https://metadados.snirh.gov.br/), and land cover data from the MapBiomas project (https://brasil.mapbiomas.org/). All spatial layers are publicly available and used under terms compatible with publication under the CC BY 4.0 license. AC: Acre; AM: Amazonas; PA: Pará; RO: Rondônia.

For each case, two environmental background locations were randomly selected within the same municipality. This procedure yielded a dataset of 576 observations, including 192 cases and 384 background locations, used for spatial modelling (Fig 1). Fig 1 illustrates the spatial distribution of cases and background locations, major river systems, the calculation of distance to the nearest river, the land cover classification used to derive landscape metrics, and the circular buffers of 3-km^2^ and 10-km^2^.

Environmental variables were extracted for all case and environmental background locations using landscape buffers at two spatial scales (3-km^2^ and 10-km^2^) (Table 2). Table 2 summarizes the distribution of these variables across cases and background locations.

**Table 2:** Descriptive statistics of environmental variables for lobomycosis cases and environmental background locations at two spatial scales (3-km^2^ and 10-km^2^). Values are presented as mean (standard deviation).

Variable	Case (3-km^2^)	Background (3-km^2^)	Case (10-km^2^)	Background (10-km^2^)
Forest (PLAND, %)	60.64 (34.29)	88.55 (23.80)	65.26 (30.36)	88.09 (22.36)
Forest (LPI, %)	53.24 (36.80)	87.03 (26.24)	54.37 (34.74)	85.60 (25.98)
Water (PLAND, %)	4.37 (6.97)	0.50 (2.93)	3.57 (5.42)	0.48 (2.19)
Water (LPI, %)	3.98 (6.61)	0.39 (2.18)	3.12 (5.16)	0.36 (1.70)
Distance to river (km)	0.97 (0.97)	1.65 (1.23)	0.97 (0.97)	1.65 (1.23)
Elevation (m)	162.27 (48.78)	195.71 (55.72)	162.27 (48.78)	195.71 (55.72)
Population density^1^ (persons per pixel)	14.37 (25.83)	0.79 (7.82)	14.37 (25.83)	0.79 (7.82)

^1^Population density was derived from gridded WorldPop data and represents modeled estimates of the number of persons per pixel; non-integer values are expected.

Model selection indicated that models including LPI received greater support than models based on PLAND at both spatial scales (Table 3). Together, the LPI models accounted for more than 90% of the total Akaike weight. These results indicate that landscape configuration metrics, which describe the spatial structure of forest and water patches, were more strongly supported than landscape composition metrics for explaining lobomycosis occurrence.

**Table 3:** Model selection for generalized additive models at two spatial scales.

Model	Scale	df	AICc^1^	ΔAICc^2^	Weight (%)^3^
LPI model	10-km^2^	24	506.1	0.0	48
LPI model	3-km^2^	24	506.4	0.3	43
PLAND model	10-km^2^	24	510.0	3.9	7
PLAND model	3-km^2^	23	512.4	6.3	2

^1^Models were ranked by AICc.

^2^Difference in AICc relative to the best-supported model.

^3^Akaike weight, expressed as the relative support for each candidate model.

Collinearity diagnostics did not indicate problematic redundancy among predictors. Variance inflation factors were low across all candidate models, ranging from 1.08 to 1.17, indicating that water-related landscape metrics and distance to rivers did not introduce substantial collinearity in the fitted models.

In the best-supported model (LPI at 10-km^2^), environmental variables showed associations with lobomycosis occurrence in directions consistent with the forest-river interface hypothesis (Table 4). Similar directions of association were observed at the 3-km^2^ scale, supporting the consistency of results across spatial scales.

**Table 4:** Associations between environmental variables and lobomycosis occurrence based on the best-supported GAM (LPI model at 10 km^2^).

Variable	OR^1^	95% CI	*p*-value
Forest (LPI, %)	0.99	0.98–0.99	0.001
Water (LPI, %)	1.19	1.07–1.33	0.002
Distance to river (km)	0.72	0.58–0.91	0.005
Elevation (m)	0.98	0.97–0.99	<0.001
Population density	1.16	1.04–1.29	0.006

^1^Odds ratio estimates are presented per one-unit increase in each variable. For LPI variables, one unit corresponds to one percentage point; distance to river is expressed in kilometers, elevation in meters, and population density as modeled persons per pixel.

Because LPI is expressed as a percentage, the forest and water LPI estimates should be interpreted as changes associated with a one-percentage-point increase in landscape dominance. Thus, the forest LPI estimate indicates a small decrease in the odds of lobomycosis occurrence for each one-percentage-point increase in forest dominance, whereas the water LPI estimate indicates an increase in the odds for each one-percentage-point increase in water-body dominance. Distance to river was interpreted per kilometer, elevation per meter, and population density per modeled person per pixel. These estimates should not be interpreted as changes over broader increments unless explicitly rescaled. No significant residual spatial autocorrelation was detected in the best-supported model (Moran’s I, *p* = 0.17).

Sensitivity analyses stratified by probable year of infection showed consistent directions of association across broad temporal periods and spatial scales (S1 Table). In both pre-2000 and post-2000 subsets, forest dominance showed negative associations with lobomycosis occurrence, water dominance showed positive associations, and distance to rivers showed negative associations. Although some associations were no longer statistically significant in the post-2000 subset, the overall direction of effects remained stable.

**Fig 2:**
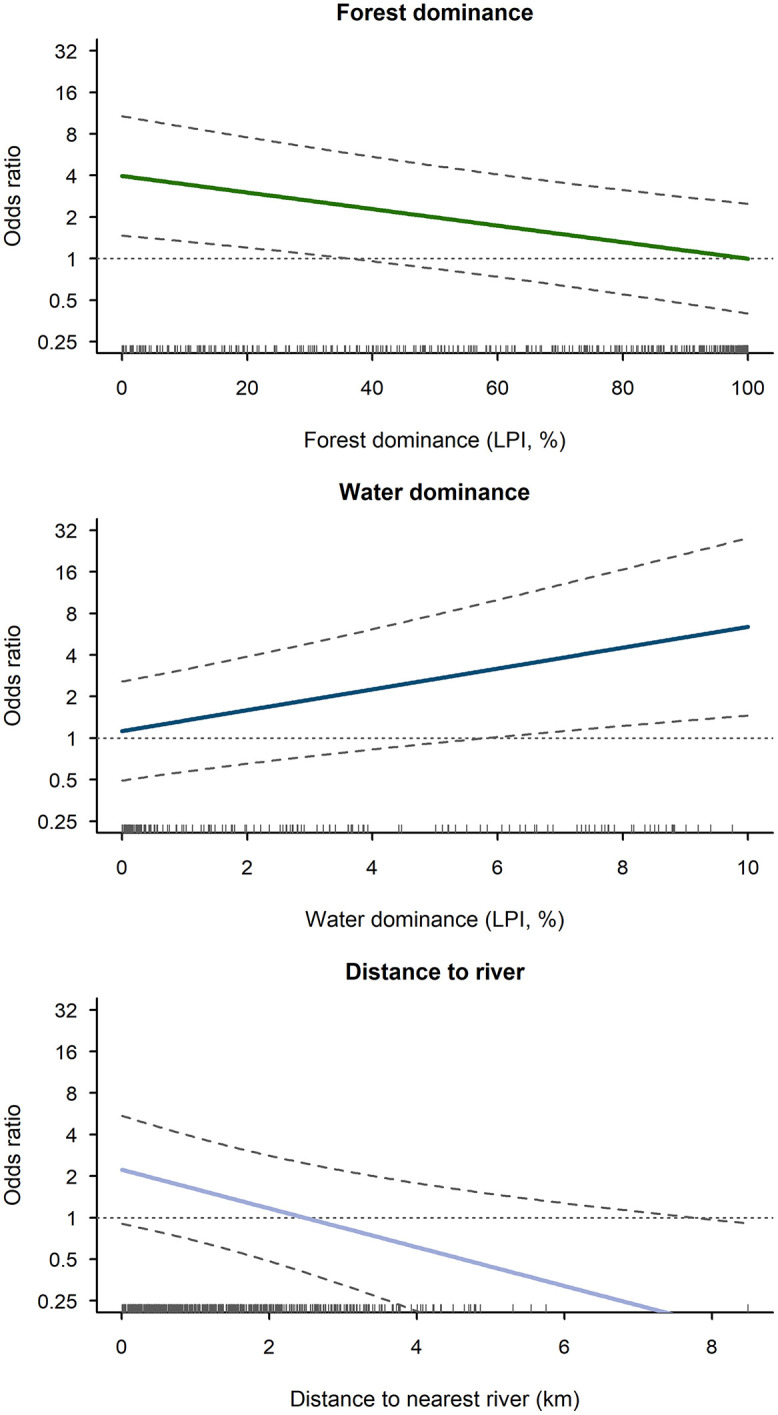
Predicted effects of key environmental variables on lobomycosis occurrence based on the best-supported GAM (LPI at 10 km^2^). Solid lines represent predicted odds ratios, and dashed lines indicate 95% confidence intervals. The horizontal dotted line indicates OR = 1. The y-axis is shown on a logarithmic scale to improve comparability across predictors. Rug plots along the x-axes show the distribution of observed values. For water dominance (LPI, %), the x-axis was truncated to 0–10% to improve visualization of the fitted effect within the ecologically relevant range supported by most observations. Estimates beyond this range should be interpreted cautiously because of sparse data and increased model uncertainty.

To further characterize these associations, predicted effects from the best-supported model were examined (Fig 2). The fitted curves indicated lower predicted odds of lobomycosis occurrence with increasing forest dominance and increasing distance to the nearest river, whereas predicted odds increased with greater water dominance. For water dominance, the relationship was strongest at low values of LPI, while predictions at higher values should be interpreted cautiously because of sparse data and greater model uncertainty.

## Discussion

This study demonstrates that the observed spatial occurrence of lobomycosis is strongly associated with landscape configuration at forest–river interfaces, rather than with forest cover alone. Models including landscape configuration metrics received greater support than models based on landscape composition. Water-body dominance and proximity to rivers were associated with lobomycosis occurrence, suggesting that the spatial arrangement of forest and aquatic environments provides relevant information for understanding where cases are observed.

The observed lobomycosis cases occurred within landscapes connected to major Amazonian river systems, particularly the Juruá, Purus, and Madeira river basins. These river corridors structure settlement, mobility, subsistence practices, and access routes across the western Amazon, and may therefore influence both environmental exposure opportunities and the spatial detection of cases. This pattern is consistent with previous reports of lobomycosis clusters associated with large river systems, including the Tapajós River in Brazil and the Orinoco River in northern South America [2,14,16].

These findings refine the traditional view of lobomycosis as a disease primarily associated with deep forest exposure. Previous epidemiological descriptions have emphasized forest-related occupations and traumatic inoculation through contact with vegetation [2–4,13–18]. Our results do not contradict this forest-associated paradigm. Instead, they suggest that lobomycosis occurrence may be concentrated where forest environments intersect with aquatic systems. In these landscapes, riparian vegetation may coincide with activities that increase opportunities for traumatic skin exposure.

The occupational classification in this study also supports this interpretation. Although forest-related activities were common, many cases occurred among individuals involved in other occupations. This pattern is consistent with the broader clinical and epidemiological literature showing that lobomycosis occurs across diverse Amazonian and tropical populations from regions beyond Brazil, including Colombia, Venezuela, Bolivia, French Guiana, and Panama [1,5,6,14,16,17].

These results are consistent with the concept that disease occurrence can emerge from ecological interfaces rather than from single environmental components alone [19]. Forest–river interfaces may represent zones where environmental suitability and human exposure opportunities overlap. Similar interface-based patterns have been described for other infectious diseases in the Amazon and tropical South America, where landscape configuration and environmental heterogeneity influence transmission dynamics [21,33,36]. In the present study, the dominance and spatial arrangement of forest and water patches were more informative than the total amount of forest or water for understanding lobomycosis occurrence.

The association with water-body dominance and proximity to rivers raises the possibility that aquatic or semi-aquatic environments contribute to the ecology of *P*. *lobogeorgii*. The environmental reservoir of the agent remains unknown, and the organism has not been cultured from environmental samples [3,4,15]. Therefore, the associations observed here should be interpreted as indirect spatial evidence rather than proof of a specific reservoir. Nevertheless, the pattern is biologically plausible. Riverine environments are characterized by high humidity, decaying organic matter, and frequent contact between humans and plants. These conditions may provide suitable habitats for fungal persistence while also increasing opportunities for traumatic inoculation during daily activities [39,40].

Several non-mutually exclusive transmission pathways may be compatible with these findings. Traumatic inoculation through plant material, thorns, or branches remains a central hypothesis, particularly in riparian environments [15,18]. Direct or indirect contact with aquatic or semi-aquatic substrates may increase opportunities for inoculation, especially during fishing or movement through flooded forest margins [39]. Arthropod-associated trauma also deserves further investigation [10]. The recent report of lobomycosis in a child with tick exposure raises the hypothesis that vector-associated mechanisms may contribute to transmission in some settings, although current evidence remains insufficient to establish ticks as biological vectors [10].

Evidence from aquatic mammals provides an additional, although indirect, reason to consider aquatic and semi-aquatic hypotheses. Lobomycosis-like disease has been described in dolphins and other small cetaceans, with cutaneous granulomatous lesions resembling human lobomycosis [39,40]. Molecular studies have since clarified that the dolphin-associated pathogen is distinct from *P*. *lobogeorgii* and is currently classified as *Paracoccidioides ceti* [41]. Although dolphin disease should not be interpreted as evidence that the same pathogen infects humans and cetaceans, it supports the broader possibility that *Paracoccidioides*-related fungi associated with chronic cutaneous disease in mammals may persist in aquatic environments [42]. This comparative evidence strengthens the rationale for investigating riparian soils, aquatic plants, and arthropods in future studies aiming to clarify the environmental reservoir of *P*. *lobogeorgii*.

Host susceptibility may be an additional component in lobomycosis pathogenesis. In other environmentally acquired fungal diseases, exposure is necessary but not sufficient for disease development, and host immune-related factors may influence susceptibility and clinical expression. Chromoblastomycosis provides a relevant comparison because it is also an implantation mycosis acquired through traumatic inoculation, and susceptibility has been linked to host-related factors, fungal traits, and environmental exposure [43]. Recent reviews of paracoccidioidomycosis, a distinct environmentally acquired systemic mycosis, suggest that immune-related genetic variation may influence susceptibility or disease progression, although evidence remains limited [44]. By analogy, lobomycosis may also depend on interactions among environmental exposure, repeated traumatic inoculation, pathogen persistence, and individual host response. We did not have immunological or genetic data to test this hypothesis. Future studies should investigate whether host factors modify the probability of developing clinically apparent lobomycosis after exposure.

Surveillance and prevention of lobomycosis in Amazonian landscapes should not be limited to individuals formally classified as rubber tappers or forest extractivists. Communities located near major rivers may require attention regardless of reported occupation. Proximity to rivers and landscape configuration could help identify areas where active case finding, clinical training, and prevention messages are most needed.

This study has several limitations. First, the comparison group consisted of environmental background locations. This is a standard approach in spatial epidemiology when true absence data are unavailable. Randomly sampled background locations represent the environmental conditions available within the same municipality where each case occurred [23–25]. The 2-km exclusion criterion reduced spatial dependence between case and background buffers and avoided overlap during landscape metric extraction.

A formal *a priori* sample size or power calculation was not performed. The analysis included all georeferenced confirmed cases available for the study area and study period, representing a substantial dataset for a rare and neglected disease such as lobomycosis. Nevertheless, the absence of a formal power calculation should be considered when interpreting the precision of estimates, particularly in subgroup analyses such as the pre-2000 and post-2000 sensitivity analyses.

The spatial distribution of observed cases may be influenced by access to specialized diagnosis. In Amazonian riverine landscapes, many communities are located far from urban referral services, sometimes requiring long boat trips over several days to reach specialized healthcare [20,22]. In the present study, this limitation was partly addressed through active field surveillance. Multidisciplinary teams visited localities across the study area to collect clinical and epidemiological information, georeference probable exposure locations, and provide local assistance to affected populations. Case detection may nevertheless remain incomplete.

Residential location at the time of lesion onset was used as the most plausible proxy for the site of infection. This assumption is unavoidable in a retrospective study of a chronic disease with slow lesion progression and potentially long incubation [3–5,14]. Riverine and rural populations often have activity spaces that extend beyond the household, including fishing sites or agricultural plots. The georeferenced residential location should therefore be interpreted as an anchor point for the likely exposure environment.

The study is ecological in nature, but it provides a finer spatial characterization than conventional municipality-level ecological analyses. Instead of assigning exposure based only on administrative units, environmental variables were extracted within 3-km^2^ and 10-km^2^ buffers around georeferenced case and background locations. These buffers represent local landscape contexts around the reported residence at lesion onset and were intended to approximate the spatial scale at which routine activities near households may occur [21]. The stronger support for the 10-km^2^ LPI model suggests that broader local landscape structure may be particularly relevant. As these metrics do not directly measure individual behavior or time spent in specific habitats, future studies should combine this refined spatial approach with individual-level exposure data and prospective clinical follow-up.

The probable year of infection was inferred from patient-reported lesion onset and clinical history, and the corresponding annual land cover map was used to characterize the landscape at that time [26]. This approach improves temporal alignment compared with using a single contemporary land cover map for all cases. Sensitivity analyses stratified by broad time periods showed consistent directions of association, although residual temporal uncertainty remains.

The environmental analysis focused on primary forest and water bodies because they were directly linked to the study hypothesis. LPI was selected to test whether dominance of primary forest, representing the traditional forest-exposure hypothesis, or dominance of water bodies, representing the forest–river interface hypothesis, was associated with lobomycosis occurrence relative to environmental background locations. Other land cover classes and metrics may provide additional information in future studies but should be tested within explicit ecological hypotheses.

Together, these findings support a conceptual shift of lobomycosis epidemiology, from a disease of forest exposure alone to one structured by ecological interfaces. Forest–river landscapes appear to be important zones where environmental suitability, human mobility, and opportunities for traumatic inoculation may overlap. These findings do not identify the environmental reservoir or prove individual transmission pathways, but they provide spatial evidence that forest–river configuration and river proximity are important features of the observed distribution of lobomycosis in the Amazon Basin. Future studies integrating environmental sampling, individual exposure assessment, host susceptibility, and pathogen detection are needed to clarify direct and indirect routes of transmission.

## Supporting information

S1 Table Sensitivity analysis stratified by probable year of infection. Odds ratios, 95% confidence intervals, and *p*-values are shown for LPI models fitted separately for pre-2000 and post-2000 observations at 3-km^2^ and 10-km^2^ scales.
(DOCX)
