# Supplementary material for: Forest–river interfaces shape lobomycosis risk in the Amazon Basin
Source: PLoS Negl Trop Dis. 2026 Jul 6;20(7):e0014502. doi: 10.1371/journal.pntd.0014502 (PMC13349294; doi:10.1371/journal.pntd.0014502)
Supplement: S1 Table — Odds ratios, 95% confidence intervals, and p-values are shown for LPI models fitted separately for pre-2000 and post-2000 observations at 3-km2 and 10-km2 scales. (DOCX) [file pntd.0014502.s001.docx]

**S1 Table. Sensitivity analysis stratified by probable year of infection.** Odds ratios, 95% confidence intervals, and *p*-values are shown for LPI models fitted separately for pre-2000 and post-2000 observations at 3-km^2^ and 10-km^2^ scales.

| **Variable** | **Scale** | **Period** | **OR** | **95% CI** | ***p*-value** |
| --- | --- | --- | --- | --- | --- |
| Forest (LPI, %) | 3-km^2^ | pre-2000 | 0.99 | 0.98–1.00 | 0.029 |
| Water (LPI, %) | 3-km^2^ | pre-2000 | 1.13 | 1.02–1.26 | 0.020 |
| Distance to river (km) | 3-km^2^ | pre-2000 | 0.61 | 0.45–0.83 | 0.001 |
| Elevation (m) | 3-km^2^ | pre-2000 | 0.98 | 0.97–0.99 | <0.001 |
| Population density | 3-km^2^ | pre-2000 | 1.13 | 1.05–1.22 | 0.002 |
| Forest (LPI, %) | 3-km^2^ | post-2000 | 0.98 | 0.97–0.99 | 0.004 |
| Water (LPI, %) | 3-km^2^ | post-2000 | 1.15 | 0.96–1.37 | 0.130 |
| Distance to river (km) | 3-km^2^ | post-2000 | 0.83 | 0.59–1.17 | 0.280 |
| Elevation (m) | 3-km^2^ | post-2000 | 0.97 | 0.96–0.99 | 0.004 |
| Population density | 3-km^2^ | post-2000 | 1.26 | 0.87–1.81 | 0.219 |
| Forest (LPI, %) | 10-km^2^ | pre-2000 | 0.99 | 0.98–1.00 | 0.127 |
| Water (LPI, %) | 10-km^2^ | pre-2000 | 1.25 | 1.09–1.44 | 0.002 |
| Distance to river (km) | 10-km^2^ | pre-2000 | 0.60 | 0.44–0.81 | 0.001 |
| Elevation (m) | 10-km^2^ | pre-2000 | 0.98 | 0.97–0.99 | <0.001 |
| Population density | 10-km^2^ | pre-2000 | 1.16 | 1.04–1.31 | 0.010 |
| Forest (LPI, %) | 10-km^2^ | post-2000 | 0.98 | 0.97–1.00 | 0.009 |
| Water (LPI, %) | 10-km^2^ | post-2000 | 1.13 | 0.94–1.36 | 0.207 |
| Distance to river (km) | 10-km^2^ | post-2000 | 0.82 | 0.58–1.17 | 0.273 |
| Elevation (m) | 10-km^2^ | post-2000 | 0.97 | 0.96–0.99 | 0.003 |
| Population density | 10-km^2^ | post-2000 | 1.30 | 0.86–1.97 | 0.211 |
