# Supplementary material for: Forest–river interfaces shape lobomycosis risk in the Amazon Basin
Source: PLoS Negl Trop Dis. 2026 Jul 6;20(7):e0014502. doi: 10.1371/journal.pntd.0014502 (PMC13349294; doi:10.1371/journal.pntd.0014502)
Supplement: Attachment — Submitted filename: Response_to_Reviewers_signed.pdf [file pntd.0014502.s003.pdf]

Response to Reviewers

Manuscript ID: PNTD-D-26-00616
Title: Forest–river interfaces shape lobomycosis risk in the Amazon Basin

Dear Editors and Reviewers,
We thank for the careful evaluation of our manuscript and for recognizing the relevance and novelty of applying spatial epidemiology to lobomycosis in the Amazon Basin. We are grateful for the constructive comments, which helped us improve methodological clarity, reproducibility, ecological interpretation, and clinical framing of the study.

In the revised manuscript, we have clarified the case–background design, expanded the description of background locations, aligned land-cover extraction with the estimated year of infection, added sensitivity analyses stratified by time period, reported collinearity diagnostics, revised the interpretation of odds ratios, improved Fig 2 with truncated water-LPI axis and rug plots, expanded Table 1 occupational categories, strengthened the Discussion regarding ecological inference, residential anchoring, human mobility, aquatic/semi-aquatic hypotheses, host susceptibility, and public health implications, and added an Author Summary. We also revised the data availability and reproducibility materials, including a confidential review dataset with spatially jittered coordinates and a native R script for reviewer/editor assessment.

Below, we provide a point-by-point response.

Responses to Additional Editor Comments

1. Control Selection and Spatial Bias

Editor comment:
Control Selection and Spatial Bias: As highlighted in my initial review and echoed by Reviewer 2, the epidemiological definition and spatial selection of your controls require clarification and potential re-analysis. By enforcing a 2km exclusion zone between cases and controls, there is a risk of artificially forcing controls into a different ecological stratum (e.g., dense forest away from rivers). Please clarify whether these controls represent true disease absence or simply environmental pseudo-absences. Furthermore, please discuss how this design choice, combined with the clustering of human settlements and healthcare access along waterways might confound your environmental associations.

Response:
We agree that the epidemiological meaning of the comparison group required clarification. We have revised the manuscript throughout to describe the design as a **spatial case–background study**, rather than a traditional individual case–control study. We now explicitly state that background locations are environmental pseudo-absence points sampled from the same municipal domain as cases, not confirmed disease-free individuals.

We clarified this in the Methods under **Study design and study area** and **Selection of background** locations. We also explained that the 2-km exclusion criterion was used to reduce spatial dependence between case and background buffers and avoid buffer overlap during landscape metric extraction. Background points were still sampled within the same municipality to represent the environmental conditions available in the same geographic domain where each case occurred.

We also expanded the Discussion to acknowledge that case detection may be influenced by settlement patterns, riverine mobility, and access to specialized diagnosis. We note that many Amazonian riverine communities are located far from referral centers and that case detection may remain incomplete, although this limitation was partly addressed through active field surveillance by multidisciplinary teams.

2. Temporal Heterogeneity and Exposure Misclassification

Editor comment:
Temporal Heterogeneity and Exposure Misclassification: Reviewer 2 highlights that the wide temporal span of your cases (pre-1979 to ≥2020) occurring in a context of rapid landscape change in the Amazon could introduce a risk of exposure misclassification. The probable year of infection metric requires further justification. As suggested, conducting sensitivity analyses stratified by time period (e.g., pre-2000 vs. post-2000) or explicitly incorporating temporal uncertainty into the GAM framework is recommended.

Response:
We agree. We have expanded the Methods to clarify how the probable year of infection was inferred from clinical history and reported lesion onset. We also clarified that annual MapBiomas land-cover maps were selected according to the estimated year of infection, thereby improving temporal alignment between environmental covariates and inferred exposure timing.

In addition, we conducted a new sensitivity analysis stratified by probable year of infection into **pre-2000** and **post-2000** periods. This analysis is described in the Methods and reported in the Results, with the full output provided as **S1 Table**. The sensitivity analysis showed consistent directions of association across broad temporal periods: forest dominance remained negatively associated with lobomycosis occurrence, water dominance remained positively associated, and distance to rivers remained negatively associated. Some associations were not statistically significant in the post-2000 subset, but the overall direction of effects remained stable.

We also added a limitation noting that residual temporal uncertainty remains, particularly because the probable year of infection was inferred from patient-reported lesion onset and clinical history.

3. Residential Anchoring and the Site of Infection

Editor comment:
Residential Anchoring and the Site of Infection: As highlighted by Reviewer 3, the long incubation period of lobomycosis complicates spatial attribution. Georeferencing cases based on residential addresses assumes the home environment is the site of exposure. However, riverine populations frequently travel into surrounding forested areas for occupational or subsistence activities. Therefore, finding that waterways and forest-river interfaces are significant predictors may simply reflect the geography of human settlement patterns (where people live) rather than the true ecological niche of the pathogen (where people are exposed). Please address the limitation of using residential coordinates as a proxy for the actual, unobserved sites of contamination, and discuss how human mobility and activity spaces might confound your environmental associations.

Response:
We agree and have revised both the Methods and Discussion to address this point explicitly. In the Methods, we now state that the reported place of residence at the time of lesion onset was used as the primary spatial reference under the assumption that it represents the most plausible proxy for the site of infection.

In the Discussion, we added a limitation explaining that residential location at lesion onset should be interpreted as an **anchor point for the likely exposure environment**, not as proof that infection occurred at the household. We also acknowledge that riverine and rural populations often have activity spaces extending beyond the household, including fishing sites and agricultural plots. Therefore, the observed associations with rivers and forest–river interfaces may reflect both environmental exposure opportunities and the spatial organization of human mobility and settlement.

4. Ecological Fallacy

Editor comment:
Ecological Fallacy: Please ensure the discussion more explicitly acknowledges the ecological nature of these findings. Environmental proxies at the 3km and 10km scale cannot be directly interpreted as individual exposure pathways.

Response:
We have revised the Discussion to explicitly state that the study is ecological in nature. We also clarified that, although the study provides a finer spatial characterization than conventional municipality-level ecological analyses, the 3-km^2 and 10-km^2 buffers summarize local landscape context and should not be interpreted as direct measurements of individual exposure pathways.

We now state that landscape metrics such as forest LPI, water LPI, and distance to rivers do not directly measure individual behavior, trauma events, water contact, forest contact, occupational intensity, or time spent in specific microhabitats. We further note that future studies should combine spatial epidemiology with individual-level exposure data and prospective clinical follow-up.

5. Clinical and Genetic Context

Editor comment:

Clinical and Genetic Context: Please address Reviewer 1's comments regarding the clinical definition of lobomycosis and the expansion of Table 1 occupational data.

Response:

We addressed both points. First, we replaced "chronic cutaneous mycosis" with "**chronic implantation mycosis**" in the Abstract and Introduction. We also revised the clinical framing to describe lobomycosis as involving cutaneous and subcutaneous manifestations.

Second, we expanded Table 1 occupational categories. The previous broad category "Other occupations" was replaced with more informative categories: agriculture and farming, domestic or household activities, urban labor or service occupations, not working, and unknown, in addition to forest-related and river-related activities.

Third, we added a new Discussion paragraph on host susceptibility. We discuss that, by analogy with other environmentally acquired fungal diseases such as chromoblastomycosis, exposure may be necessary but not sufficient for disease development, and host factors may modify susceptibility. We also mention paracoccidioidomycosis as a distinct systemic mycosis in which immune-related genetic variation has been studied. We emphasize that we did not have immunological or genetic data to test this hypothesis and propose this as a direction for future research.

6. Fig 2 and Model Instability

Editor comment:

Fig 2 and Model instability: To improve the interpretability of Figure 2, please constrain the x-axis for water dominance (LPI) to an ecologically meaningful range (e.g., 0–10%) so the effect size is visible. However, you should include rug plots along the x-axis to demonstrate data density. Furthermore, please explicitly state in the figure legend that the axis has been truncated and acknowledge the model uncertainty and data sparsity beyond this threshold.

Response:

We revised Fig 2 accordingly. The water dominance x-axis is now truncated to 0–10% to improve visualization of the fitted effect within the range supported by most observations. Rug plots were added to all x-axes to show the distribution of observed values. The revised Fig 2 legend explicitly states that the water-LPI axis was truncated and that estimates beyond this range should be interpreted cautiously because of sparse data and increased model uncertainty.

7. Missing Coordinates and Code Format

Editor comment:

Missing Coordinates: The README notes that Lat/Long coordinates are set to NA for confidentiality. However, the mgcv::gam models require these coordinates for the spatial smooth term s(Lat, Long). The code will fail to execute. Please provide spatially jittered coordinates that protect patient privacy whilst allowing the spatial autocorrelation and GAM smooths to be reproduced.

Code Format: Please upload the script as a native .R or .Rmd file to zenodo.

Response:

We agree that reproducibility required improvement. To support peer review and reproducibility while protecting participant confidentiality, we uploaded the following files as Other / NOT FOR PUBLICATION for editor and reviewer assessment only:

- Confidential_review_dataset_10km_jittered_coordinates_NOT_FOR_PUBLICATION.csv
- Reviewer_reproducibility_script_10km.R
- README_reviewer_reproducibility_NOT_FOR_PUBLICATION.txt

The review dataset includes anonymized case–background observations, derived environmental variables, and spatially jittered coordinates. The R script is provided in native .R format and reproduces the main 10-km^2 analytical workflow, including GAM fitting with the spatial smooth term, model comparison, odds ratios, confidence intervals, VIF diagnostics, temporal sensitivity analysis, and a Fig 2-style prediction plot.

Because residual spatial autocorrelation diagnostics depend directly on the original pairwise spatial distances, Moran's I was not recalculated in the reproducibility script using jittered coordinates. The Moran's I result reported in the manuscript was calculated using the original confidential coordinates. We explain this in the README file.

We have also revised the **Data availability** section to state that review files are provided for editors and reviewers at this stage, and that anonymized reproducibility files, spatially jittered coordinates, derived environmental variables, and native R code will be deposited in Zenodo upon acceptance.

8. Base Map Licensing

Editor comment:

Base Map Licensing: Please ensure that all base layers and shapefiles used in Figure 1 are compatible with PLOS's CC BY 4.0 open-access license. Cite the open-source origin of these layers (e.g., MapBiomas, OpenStreetMap) in the figure legend.

Response:

We revised the Fig 1 legend to identify the sources of all spatial layers used in the map and to provide direct links to the corresponding base layers. The revised legend now states that South American country boundaries were obtained from Natural Earth Admin 0 – Countries, Natural Earth Ocean for ocean polygons, Brazilian administrative boundaries from the Brazilian Institute of Geography and Statistics territorial meshes, hydrographic layers from the Brazilian National Water Agency, and land-cover data from the MapBiomas project. We also state in the legend that all spatial layers are publicly available and used under terms compatible with publication under the CC BY 4.0 license.

9. Journal Requirements

Editor comment:

Please provide an Author Summary; upload main figures as separate .tif or .eps files; and provide a link/source/license for the base layer of Fig 1.

Response:

We added an **Author Summary** between the Abstract and Introduction. We also revised the Abstract headings to follow PLOS NTD format: Background, Methodology/Principal Findings, and Conclusions/Significance. Main figures will be uploaded as separate figure files in accordance with PLOS requirements. The Fig 1 legend now identifies the open-source origins of the spatial layers and confirms compatibility with CC BY 4.0 publication requirements.

Responses to Reviewer #1

Comment 1. Occupational data in Table 1

Reviewer comment:

In Table 1, most patients are grouped under 'other occupations' in terms of their work activities. However, this classification is too nonspecific. Given the relevance of occupational data, this category should be further clarified and broken down into more informative subgroups.

Response:

We agree. We revised Table 1 to break down the previous broad "Other occupations" category into more informative subgroups. The revised occupational categories are:

- Forest-related activities
- River-related activities
- Agriculture and farming
- Domestic or household activities
- Urban labor or service occupations
- Not working
- Unknown

This provides a more transparent description of the occupational profile of the study population and supports the Discussion that lobomycosis occurrence is not restricted to individuals formally classified as forest extractivists or fishermen.

Comment 2. Genetic susceptibility

Reviewer comment:

Previous studies suggest that in chromoblastomycosis and paracoccidioidomycosis, there is some evidence that genetic susceptibility may influence the development of clinical manifestations in patients exposed to sources of infection. This helps explain why, although these agents live saprobiotically in several environmental sources of infection in nature, only a small number of individuals become ill. Do you think that patients with lobomycosis may develop disease due to some form of genetic susceptibility?

Response:

We thank the reviewer for this important comment. We added a paragraph to the Discussion addressing host susceptibility as a possible component of lobomycosis pathogenesis. We state that, in environmentally acquired fungal diseases, exposure may be necessary but not sufficient for disease development. We discuss chromoblastomycosis as a relevant comparison because it is also an implantation mycosis acquired through traumatic inoculation, and susceptibility has been linked to host-related factors, fungal traits, and environmental exposure. We also mention paracoccidioidomycosis as a distinct systemic mycosis in which immune-related genetic variation has been studied.

We make clear that we did not have immunological or genetic data to test this hypothesis in the present study. We therefore propose that future studies should evaluate whether host factors modify the probability of developing clinically apparent lobomycosis after exposure.

Comment 3. Use of "implantation mycosis"

Reviewer comment:

Lobomycosis is an implantation mycosis, and it should not be defined as a 'chronic cutaneous mycosis' like dermatophytosis or dermatomycosis. Patients with lobomycosis may present with a wide spectrum of cutaneous and subcutaneous manifestations...

Response:

We agree and have revised the manuscript accordingly. We replaced "chronic cutaneous mycosis" with "**chronic implantation mycosis**" in the Abstract and Introduction. We also revised the clinical description to refer to slowly progressive cutaneous and subcutaneous lesions. This terminology better reflects the pathogenesis and clinical spectrum of lobomycosis.

Responses to Reviewer #2

Comment 1. Temporal heterogeneity and exposure misclassification

Reviewer comment:

The main concern relates to the time span of the 192 cases... This temporal heterogeneity may introduce exposure misclassification bias... Suggestion: perform sensitivity analyses stratified by time period (e.g., pre-2000 vs post-2000) or restrict analyses to a subset with higher temporal reliability. Alternatively, incorporate temporal uncertainty into the modeling framework.

Response:

We agree and have revised the manuscript accordingly. The Methods now provide a clearer explanation of how the probable year of infection was inferred from patient-reported lesion onset and clinical history. We also clarified that the corresponding annual MapBiomas land-cover map was selected for each case, aligning landscape characterization with the inferred exposure period.

We conducted new sensitivity analyses stratified by probable year of infection into pre-2000 and post-2000 periods. These analyses are described in the Methods, summarized in the Results, and reported in S1 Table. The analyses showed consistent directions of association across broad temporal periods and spatial scales, although some post-2000 estimates were not statistically significant. We also discuss residual temporal uncertainty as a limitation.

Comment 2. Definition and epidemiological meaning of controls

Reviewer comment:

Although described as 'randomly selected within the same municipality', it is not entirely clear whether they represent true absence of disease or simply absence of recorded cases. Moreover, there is no guarantee that these locations correspond to unexposed populations. Suggestion: clarify the epidemiological rationale for control selection.

Response:

We agree. We revised the terminology throughout the manuscript from "controls" to "**environmental background locations**" and describe the study as a **spatial case–background study**. We now explicitly state that background locations are environmental pseudo-absence points and not confirmed disease-free individuals.

The Methods now explain that background points represent the environmental conditions available within the same municipality where each case occurred. The Discussion also acknowledges that this design compares observed case locations with the broader environmental background and should not be interpreted as estimating individual-level risk from population-based controls.

Comment 3. Matching and intra-municipality heterogeneity

Reviewer comment:

While controls are 'landscape-matched' at the municipal level, there is no individual matching, and municipalities in the Amazon can be highly heterogeneous. This may result in residual confounding. Suggestion: consider models with random effects at the municipality level or conditional analyses to better account for intra-municipality variability.

Response:

We clarified in the Methods that background locations were restricted to the same municipality to ensure that comparisons occurred within the same broad geographic domain, but that they were not individually matched in the statistical models. We also revised the terminology to avoid implying individual matching.

We accounted for spatial structure using a two-dimensional smooth term of geographic coordinates in the GAMs. We also evaluated residual spatial autocorrelation using Moran's I. We agree that municipality-level heterogeneity remains a potential source of residual confounding, and this is now better acknowledged in the Discussion through the limitations regarding ecological inference, spatial detection, and activity-space interpretation.

Comment 4. Georeferencing uncertainty

Reviewer comment:

The georeferencing process is a methodological strength... Nevertheless, spatial error remains possible, particularly for older cases... Suggestion: explicitly discuss the expected magnitude of spatial error and, if possible, conduct sensitivity analyses using larger buffers or spatial jittering.

Response:

We expanded the Methods to clarify that cases were georeferenced using the highest spatial precision achievable from clinical records, active case investigation, contact with local health services, field verification, and GPS coordinates when available. We also added a Discussion paragraph noting that residential location at lesion onset is a proxy for the likely exposure environment, particularly in a chronic disease with slow progression and potentially long incubation.

The analyses already used two spatial scales, 3-km^2 and 10-km^2, which provides a spatial-scale sensitivity component. The stronger support for the 10-km^2 LPI model is now discussed as suggesting that broader local landscape structure may be particularly relevant. For reproducibility and confidentiality, we also provide spatially jittered coordinates in the confidential review dataset.

Comment 5. Environmental simplification and additional land-cover classes

Reviewer comment:

The analysis may be overly simplified by considering only two land cover classes... Suggestion: incorporate additional metrics or intermediate land cover classes.

Response:

We have clarified the rationale for focusing on primary forest and water bodies. The environmental analysis focused on these classes because they were directly linked to the study hypothesis: primary forest represents the traditional forest-exposure hypothesis, whereas water bodies represent the alternative forest–river interface hypothesis. LPI was selected to test whether dominance of either class was associated with lobomycosis occurrence relative to environmental background locations.

We agree that other classes and metrics may provide additional information. We therefore added a Discussion statement noting that anthropogenic land-cover classes, edge metrics, and additional landscape metrics may be valuable in future studies, but should be tested within explicit ecological hypotheses rather than added as exploratory predictors in the same model.

Comment 6. Buffer size justification

Reviewer comment:

The choice of buffer sizes (3 km^2 and 10 km^2) is reasonable but lacks strong epidemiological justification... Suggestion: test additional spatial scales or apply a continuous multi-scale approach.

Response:

We expanded the Methods and Discussion to better justify the buffer scales. We now state that 3-km^2 and 10-km^2 buffers were selected to capture two complementary local spatial contexts: the immediate surroundings of the georeferenced location and the broader landscape context potentially associated with fishing, forest use, agricultural activities, and riverine mobility. We also clarify in the Discussion that these buffers provide a finer spatial characterization than conventional municipality-level ecological analyses, while acknowledging that they are still ecological proxies and do not directly measure individual activity spaces.

Comment 7. Collinearity diagnostics

Reviewer comment:

Including both distance to rivers and water-related landscape metrics (LPI) may introduce collinearity... Suggestion: provide collinearity diagnostics.

Response:

We added collinearity diagnostics to the Methods and Results. Variance inflation factors were calculated from equivalent parametric model structures including the same predictors but excluding the spatial smooth term.

The Results now state that VIF values were low across all candidate models, ranging from 1.08 to 1.17, indicating no substantial collinearity between water-related landscape metrics and distance to rivers.

Comment 8. Odds ratio scaling and interpretation

Reviewer comment:

Interpretation of odds ratios also requires caution. For instance, the reported effect of water (LPI) as a '5-fold higher odds' per 10% increase appears exaggerated and may reflect scaling or nonlinear effects.

Response:

We agree and have revised the Results to avoid overinterpretation. Table 4 now reports ORs per one-unit increase in each variable, and the footnote clarifies that one unit of LPI corresponds to one percentage point. We removed the previous "5-fold higher odds" interpretation. The Results now explicitly state that ORs for forest and water LPI should be interpreted as changes associated with a one-percentage-point increase in landscape dominance.

Comment 9. Ethical aspects and spatial anonymization

Reviewer comment:

An important concern relates to retrospective geolocation data and the potential for indirect identification of vulnerable populations. While coordinate masking is mentioned, the method is not detailed. Suggestion: explicitly describe the spatial anonymization procedure and discuss residual re-identification risks.

Response:

We revised the Data Availability section to clarify that original geographic coordinates are not shared to protect participant confidentiality. For peer review, we provide an anonymized 10-km^2 analytical dataset with spatially jittered coordinates and a native R script as review files for editors and reviewers only. The README accompanying these files states that direct identifiers, locality labels, and nearest-river names were removed, and that coordinates were spatially jittered to reduce re-identification risk.

Upon acceptance, anonymized reproducibility files, derived environmental variables, spatially jittered coordinates, and native R code will be deposited in Zenodo consistent with PLOS data availability requirements and participant confidentiality protections.

Comment 10. Ecological inference

Reviewer comment:

Finally, a major limitation is ecological inference... Suggestion: emphasize the ecological nature of the findings and propose future studies incorporating individual-level exposure data.

Response:

We revised the Discussion to explicitly acknowledge the ecological nature of the study. We now state that the 3-km^2 and 10-km^2 buffers summarize local landscape context and cannot be interpreted as direct measures of individual exposure pathways. We further state that these metrics do not directly measure individual behavior or time spent in specific habitats. We propose that future studies combine refined spatial epidemiological approaches with individual-level exposure data, environmental sampling, host susceptibility assessment, and pathogen detection.

Comment 11. Model diagnostics and modeling decisions

Reviewer comment:

Transparency could be further enhanced by briefly summarizing key modeling decisions... and model diagnostics in the main text.

Response:

We added model diagnostics and modeling decisions in the main text. Specifically, the revised Methods and Results now include AICc-based model comparison, collinearity assessment using VIF, residual spatial autocorrelation assessment using Moran's I, and sensitivity analyses stratified by probable year of infection.

Comment 12. Figure and visual presentation

Reviewer comment:

For the statistical figures, adding rug plots or data density indicators would help readers assess the distribution of observations across the range of predictors.

Response:

We revised Fig 2 to include rug plots along the x-axes. We also truncated the water LPI axis to 0–10%, following the editor's recommendation, and revised the legend to explicitly state that the axis was truncated and that predictions beyond this range should be interpreted cautiously due to sparse data and increased model uncertainty.

Comment 13. Aquatic animals and dolphins

Reviewer comment:

The discussion would benefit from integrating evidence of lobomycosis-like disease in aquatic animals, particularly dolphins...

Response:

We expanded the Discussion to include evidence from aquatic mammals. We discuss lobomycosis-like disease in dolphins and other small cetaceans and note that molecular studies have clarified that the dolphin-associated pathogen is distinct from *P. lobogeorgii* and is currently classified as *Paracoccidioides ceti*. We emphasize that dolphin disease should not be interpreted as evidence that the same pathogen infects humans and cetaceans, but that it supports the broader plausibility of aquatic or semi-aquatic environments in the ecology of *Paracoccidioides-related* fungi.

Responses to Reviewer #3

Comment 1. Geographic distribution in the Introduction

Reviewer comment:

In the introduction, please do not limit the spread of Lobomycosis to Brazil and Peru. Notably, please mention Jose A Suárez Emerg Infect Dis. 2023 regarding Panama, and Grotta et al. J Fungi 2023.

Response:

We agree and revised the Introduction accordingly. The revised text now states that lobomycosis occurs mainly in tropical regions of the Americas, with reports from South America and Central America, including the Amazon Basin, Panama, and French Guiana. We added and cited the suggested references by Suárez et al. and Grotta et al.

Comment 2. Environmental routes, animal vectors, and tick exposure

Reviewer comment:

Third paragraph of the introduction, regarding environmental routes and risks, please mention possible animal vectors and notably cite Gonçalves et al. Emerg Infect Dis. 2025.

Response:

We revised the Introduction to mention animal-associated exposures and possible arthropod-associated trauma. We cite the reported pediatric case with tick exposure and state that this raises the hypothesis that arthropod-associated trauma, or potentially vector-associated mechanisms, may contribute to transmission in some settings. We also revisit this hypothesis in the Discussion, while emphasizing that current evidence is insufficient to establish ticks as biological vectors.

Comment 3. Discrepancies between addresses and presumed places of contamination

Reviewer comment:

In the methods, how did the authors manage the possible discrepancies between the patients' addresses and the presumed places of contamination? Did they always match? The incubation can be long and determining the place of contamination can be associated with bias.

Response:

We agree that this issue required explicit clarification. In the Methods, we now state that patients were asked during clinical anamnesis about the approximate year of lesion onset and place of residence at that time. The reported residence at lesion onset was used as the primary spatial reference for georeferencing, under the assumption that it represents the most plausible proxy for the site of infection.

We also added a limitation in the Discussion noting that the true site of infection was unobserved and that riverine and rural populations often have activity spaces extending beyond the household. Therefore, the georeferenced residential location should be interpreted as an anchor point for the likely exposure environment rather than as proof that infection occurred at the household itself.

Comment 4. Discussion redundancy and interpretation of water-associated hypotheses

Reviewer comment:

The discussion could be partly rewritten. There are redundancies... How do we interpret these results, regarding possible water-associated hypotheses? The authors should give hints about possible future studies and research consequences regarding the different direct and indirect routes of infection.

Response:

We substantially revised the Discussion to remove redundancy and improve interpretation. We now discuss several non-mutually exclusive transmission hypotheses, including traumatic inoculation through plant material, contact with aquatic or semi-aquatic substrates, arthropod-associated trauma, and indirect evidence from aquatic mammals. We also discuss future studies involving riparian soils, aquatic plants, arthropods, animal hosts, host susceptibility, environmental sampling, and pathogen detection.

Additional manuscript and submission changes

In addition to the responses above, we made the following changes:

1. Added an **Author Summary** in accordance with PLOS NTD requirements.
2. Revised Abstract headings.
3. Revised Fig 1 legend to include spatial data sources, licensing compatibility, and direct links to the base map layers used in the figure.
4. Revised Fig 2 with rug plots and a truncated water-LPI axis.
5. Added Supporting Information caption for **S1 Table**.
6. Uploaded a confidential review dataset and native R script under "Other / NOT FOR PUBLICATION" to support reproducibility during peer review.
7. Updated the Data Availability statement to reflect the current review-stage data-sharing approach and planned Zenodo deposition upon acceptance.

We thank the editors and reviewers again for their constructive comments. We believe the revised manuscript is substantially improved and more transparent in its methodology, interpretation, reproducibility, and limitations.

Sincerely,

Gabriel Z.
Laporta

Assinado de forma digital por Gabriel Z. Laporta
DN: cn=Gabriel Z. Laporta, o, ou,
email=gabriel.laporta@fmabc.br, c=BR
Dados: 2026.05.14 09:15:50 -03'00'

Gabriel Z. Laporta, on behalf of all authors.
