# Supplementary material for: Forest–river interfaces shape lobomycosis risk in the Amazon Basin
Source: PLoS Negl Trop Dis. 2026 Jul 6;20(7):e0014502. doi: 10.1371/journal.pntd.0014502 (PMC13349294; doi:10.1371/journal.pntd.0014502)
Supplement: Attachment — Submitted filename: Response_to_Reviewers_signed_auresp_2.pdf [file pntd.0014502.s004.pdf]

Response to Reviewers

Manuscript ID: PNTD-D-26-00616R1
Title: Forest–river interfaces shape lobomycosis risk in the Amazon Basin

Dear Dr. Simons and Editors,
We thank for the positive assessment of our revised manuscript and for recognizing that the revisions strengthened the study. We are grateful for the opportunity to submit this final revised version.

We have addressed the remaining minor editorial points in the revised manuscript. Specifically, we clarified the scaling and interpretation of the odds ratios in Table 4 and in the Results section, added the absence of a formal a priori sample size or power calculation as a methodological limitation in the Discussion, revised Fig 2, and updated the Data Availability statement and Zenodo repository.

Below, we provide a point-by-point response.

Responses to Additional Editor Comments

1. Odds ratio scaling and interpretation

Editor comment:
Please carefully review the odds ratios reported in Table 4 alongside your textual interpretation of these values in the Results section. There appear to be potential inconsistencies between the statistical output and the narrative description of the effect sizes. Please ensure the scaling and magnitude of these effects are described accurately and without overestimation.

Response:
We agree and have revised the Results section and Table 4 footnote to clarify the scale of each odds ratio estimate. The revised Table 4 footnote now states that odds ratio estimates are presented per one-unit increase in each variable; for LPI variables, one unit corresponds to one percentage point; distance to river is expressed in kilometers; elevation in meters; and population density as modeled persons per pixel.

We also revised the paragraph following Table 4 to clarify that the forest and water LPI estimates should be interpreted as changes associated with a one-percentage-point increase in landscape dominance. We explicitly state that these estimates should not be interpreted as changes over broader increments unless explicitly rescaled. This revision avoids overestimation of effect sizes and aligns the textual interpretation with the statistical output.

2. Absence of formal power analysis or sample size calculation

Editor comment:
While you have adequately addressed the temporal uncertainty of the retrospective design, please explicitly add the absence of a formal power analysis/sample size calculation as a methodological limitation in the Discussion section.

Response:
We have added this limitation to the Discussion. The revised text states that a formal a priori sample size or power calculation was not performed. We also clarify that the analysis included all georeferenced confirmed cases available for the study area and study period, representing a substantial dataset for a rare and neglected disease such as lobomycosis. Nevertheless, we now explicitly state that the absence of a formal power calculation should be considered when interpreting the precision of estimates, particularly in subgroup analyses such as the pre-2000 and post-2000 sensitivity analyses.

Journal Requirement: Data availability

Journal comment:
The journal strongly recommends depositing data before acceptance and notes that the minimal dataset will need to be made freely accessible upon acceptance, except where public deposition would breach compliance with the approved research ethics protocol.

Response:
We have updated the Data Availability statement and Zenodo repository. Following consultation regarding the ethical status of the spatial data, we deposited the analytical datasets with georeferenced coordinates representing the probable location of infection, together with the derived environmental variables and the full native R analysis script. These coordinates represent probable locations of infection inferred from clinical and epidemiological investigation and do not represent participants' current residential addresses. Direct personal identifiers were removed before public deposition.

The revised Data Availability statement now states that the analytical datasets underlying the findings of this study, including georeferenced coordinates representing the probable location of infection, derived environmental variables, and the full native R analysis script, are available in Zenodo. The Zenodo DOI has also been updated in the References.

Additional revision: Fig 2

Reviewer #2 comment:
Figure 2 could be visually improved for publication, and minor formatting adjustments in tables and figure legends should be considered.

Response:
We revised Fig 2 to improve visual clarity and comparability across predictors. The y-axis is now shown on a logarithmic scale, a horizontal reference line indicating OR = 1 has been added, and the solid lines representing predicted odds ratios are colored consistently with the environmental themes used in Fig 1. The confidence intervals remain shown as dashed lines, and rug plots are retained along the x-axes. The figure legend was updated to indicate that the y-axis is shown on a logarithmic scale and that the horizontal dotted line indicates OR = 1.

Additional revision: References

Response:
We reviewed and adjusted the reference list for consistency with PLOS style. In particular, we updated the Zenodo reference to the new DOI, formatted online resources more consistently, and standardized several journal abbreviations.

We thank the editors and reviewer again for their constructive evaluation. We believe that the manuscript now addresses all remaining editorial points and is ready for publication consideration.

Sincerely,

Gabriel Z. Laporta

Assinado de forma digital por Gabriel Z. Laporta
DN: cn=Gabriel Z. Laporta, o, ou, email=gabriel.laporta@fmabc.br, c=BR
Dados: 2026.06.18 14:48:48 -03'00'

Gabriel Z. Laporta, PhD
On behalf of all co-authors
