# Supplementary material for: Forest–river interfaces shape lobomycosis risk in the Amazon Basin
Source: PLoS Negl Trop Dis. 2026 Jul 6;20(7):e0014502. doi: 10.1371/journal.pntd.0014502 (PMC13349294; doi:10.1371/journal.pntd.0014502)
Supplement: Attachment — Submitted filename: Lobomycosis Comments.docx [file pntd.0014502.s002.docx]

Forest-river interfaces shape lobomycosis risk in the Amazon Basin

The commentary underscores the study’s relevance in demonstrating how spatial epidemiological data, combined with geoprocessing and remote-sensing approaches, can be used to characterize lobomycosis risk within the Amazonian endemic setting. In line with prior literature, the findings support associations with humid, forested environments and with riverine, forest-dependent populations, and they suggest comparatively higher risk at forest–river interfaces. The principal contribution highlighted by the commentary is the proposition that spatial risk is more strongly shaped by landscape configuration—specifically, the spatial arrangement of forest and water bodies at these interfaces—than by forest cover extent alone.

More broadly, the results and conclusions are consistent with previously reported epidemiological patterns for lobomycosis in the region, reinforcing established links between disease occurrence and environmental and land-use conditions characteristic of Amazonian riverine landscapes.

Major comments - “In Table 1, most patients are grouped under ‘other occupations’ in terms of their work activities. However, this classification is too nonspecific. Given the relevance of occupational data, this category should be further clarified and broken down into more informative subgroups.”

Previous studies suggest that in chromoblastomycosis and paracoccidioidomycosis, there is some evidence that genetic susceptibility may influence the development of clinical manifestations in patients exposed to sources of infection. This helps explain why, although these agents live saprobiotically in several environmental sources of infection in nature, only a small number of individuals become ill. Do you think that patients with lobomycosis may develop disease due to some form of genetic susceptibility?”

Minor comment – Lobomycosis is an implantation mycosis, and it should not be defined as a “chronic cutaneous mycosis” like dermatophytosis or dermatomycosis. Patients with lobomycosis may present with a wide spectrum of cutaneous and subcutaneous manifestations. The initial lesion may start as an indolent papule that may evolve to nodules, plaques, dyschromic macules, gummas, verruciform plaques, ulcers, scarring lesions, or keloid-like nodules. Some infiltrative lobomycotic lesions may be similar to burn scars or even tuberculoid leprous lesions.  The pinna is affected frequently as well as the lower and upper limbs. Although mucosal involvement has not been described, 10 to 25% of patients may present regional lymph adenomegaly due to lymphatic spreading of the infection.
