# Supplementary material for: Forest–river interfaces shape lobomycosis risk in the Amazon Basin
Source: PLoS Negl Trop Dis. 2026 Jul 6;20(7):e0014502. doi: 10.1371/journal.pntd.0014502 (PMC13349294; doi:10.1371/journal.pntd.0014502)
Supplement: Peer review history

# Peer review: Decision Letter 0

**DOI:** 10.1371/journal.pntd.0014502.r001

**Authors:** Marcio L Rodrigues, David Simons

24 Apr 2026

PNTD-D-26-00616

Forest-river interfaces shape lobomycosis risk in the Amazon Basin

PLOS Neglected Tropical Diseases

Dear Dr. Laporta,

Thank you for submitting your manuscript to PLOS Neglected Tropical Diseases. After careful consideration, we feel that it has merit but does not fully meet PLOS Neglected Tropical Diseases's publication criteria as it currently stands. Therefore, we invite you to submit a revised version of the manuscript that addresses the points raised during the review process.

Please submit your revised manuscript within by Jun 23 2026 11:59PM. If you will need more time than this to complete your revisions, please reply to this message or contact the journal office at plosntds@plos.org. When you're ready to submit your revision, log on to https://www.editorialmanager.com/pntd/ and select the 'Submissions Needing Revision' folder to locate your manuscript file.

Please include the following items when submitting your revised manuscript:

* A letter that responds to each point raised by the editor and reviewer(s). You should upload this letter as a separate file labeled 'Response to Reviewers'. This file does not need to include responses to any formatting updates and technical items listed in the 'Journal Requirements' section below.

* A marked-up copy of your manuscript that highlights changes made to the original version. You should upload this as a separate file labeled 'Revised Manuscript with Track Changes'.

* An unmarked version of your revised paper without tracked changes. You should upload this as a separate file labeled 'Manuscript'.

If you would like to make changes to your financial disclosure, competing interests statement, or data availability statement, please make these updates within the submission form at the time of resubmission. Guidelines for resubmitting your figure files are available below the reviewer comments at the end of this letter.

We look forward to receiving your revised manuscript.

Kind regards,

David Simons

Academic Editor

PLOS Neglected Tropical Diseases

Marcio Rodrigues

Section Editor

PLOS Neglected Tropical Diseases

Shaden Kamhawi

co-Editor-in-Chief

PLOS Neglected Tropical Diseases

orcid.org/0000-0003-4304-636XX

Paul Brindley

co-Editor-in-Chief

PLOS Neglected Tropical Diseases

orcid.org/0000-0003-1765-0002

**Additional Editor Comments:**

As you will see from the appended comments, the reviewers recognise the study as a highly relevant and innovative application of spatial epidemiology to a neglected mycosis. We appreciate the conceptual shift toward investigating forest-river interfaces.

Based on the reviews and my own assessment of the study design, the manuscript is being returned to you for major revisions before it can be considered for publication. While you should address all reviewer comments point-by-point, I would like to draw your attention specifically to the methodological concerns raised by the reviewers and myself, which should be comprehensively addressed in your revision:

Control Selection and Spatial Bias: As highlighted in my initial review and echoed by Reviewer 2, the epidemiological definition and spatial selection of your controls require clarification and potential re-analysis. By enforcing a 2km exclusion zone between cases and controls, there is a risk of artificially forcing controls into a different ecological stratum (e.g., dense forest away from rivers). Please clarify whether these controls represent true disease absence or simply environmental pseudo-absences. Furthermore, please discuss how this design choice, combined with the clustering of human settlements and healthcare access along waterways might confound your environmental associations.

Temporal Heterogeneity and Exposure Misclassification: Reviewer 2 highlights that the wide temporal span of your cases (pre-1979 to ≥2020) occurring in a context of rapid landscape change in the Amazon could introduce a risk of exposure misclassification. The probable year of infection metric requires further justification. As suggested, conducting sensitivity analyses stratified by time period (e.g., pre-2000 vs. post-2000) or explicitly incorporating temporal uncertainty into the GAM framework is recommended.

Residential Anchoring and the Site of Infection: As highlighted by Reviewer 3, the long incubation period of lobomycosis complicates spatial attribution. Georeferencing cases based on residential addresses assumes the home environment is the site of exposure. However, riverine populations frequently travel into surrounding forested areas for occupational or subsistence activities. Therefore, finding that waterways and forest-river interfaces are significant predictors may simply reflect the geography of human settlement patterns (where people live) rather than the true ecological niche of the pathogen (where people are exposed). Please address the limitation of using residential coordinates as a proxy for the actual, unobserved sites of contamination, and discuss how human mobility and activity spaces might confound your environmental associations.

Ecological Fallacy: Please ensure the discussion more explicitly acknowledges the ecological nature of these findings. Environmental proxies at the 3km and 10km scale cannot be directly interpreted as individual exposure pathways.

Clinical and Genetic Context: Please address Reviewer 1's comments regarding the clinical definition of lobomycosis and the expansion of Table 1 occupational data.

Fig 2 and Model instability: To improve the interpretability of Figure 2, please constrain the x-axis for water dominance (LPI) to an ecologically meaningful range (e.g., 0–10%) so the effect size is visible. However, you should include rug plots along the x-axis to demonstrate data density. Furthermore, please explicitly state in the figure legend that the axis has been truncated and acknowledge the model uncertainty and data sparsity beyond this threshold.

While providing your data and code is commendable, the current format is not fully reproducible:

Missing Coordinates: The README notes that Lat/Long coordinates are set to NA for confidentiality. However, the mgcv::gam models require these coordinates for the spatial smooth term s(Lat, Long). The code will fail to execute. Please provide spatially jittered coordinates that protect patient privacy whilst allowing the spatial autocorrelation and GAM smooths to be reproduced.

Code Format: Please upload the script as a native .R or .Rmd file to zenodo.

Base Map Licensing: Please ensure that all base layers and shapefiles used in Figure 1 are compatible with PLOS’s CC BY 4.0 open-access license. Cite the open-source origin of these layers (e.g., MapBiomas, OpenStreetMap) in the figure legend.

**Journal Requirements:**

1) Please provide an Author Summary. This should appear in your manuscript between the Abstract (if applicable) and the Introduction, and should be 150-200 words long. The aim should be to make your findings accessible to a wide audience that includes both scientists and non-scientists. Sample summaries can be found on our website under Submission Guidelines:

https://journals.plos.org/plosntds/s/submission-guidelines#loc-parts-of-a-submission

2) Please upload all main figures as separate Figure files in .tif or .eps format. For more information about how to convert and format your figure files please see our guidelines:

https://journals.plos.org/plosntds/s/figures

3) Figure 1: please (a) provide a direct link to the base layer of the map (i.e., the country or region border shape) and ensure this is also included in the figure legend; and (b) provide a link to the terms of use / license information for the base layer image or shapefile. We cannot publish proprietary or copyrighted maps (e.g. Google Maps, Mapquest) and the terms of use for your map base layer must be compatible with our CC BY 4.0 license.

Note: if you created the map in a software program like R or ArcGIS, please locate and indicate the source of the basemap shapefile onto which data has been plotted.

If your map was obtained from a copyrighted source please amend the figure so that the base map used is from an openly available source. Alternatively, please provide explicit written permission from the copyright holder granting you the right to publish the material under our CC BY 4.0 license.

If you are unsure whether you can use a map or not, please do reach out and we will be able to help you. The following websites are good examples of where you can source open access or public domain maps:

* U.S. Geological Survey (USGS) - All maps are in the public domain. (http://www.usgs.gov)

* PlaniGlobe - All maps are published under a Creative Commons license so please cite “PlaniGlobe, http://www.planiglobe.com, CC BY 2.0” in the image credit after the caption. (http://www.planiglobe.com/?lang=enl)

* Natural Earth - All maps are public domain. (http://www.naturalearthdata.com/about/terms-of-use/)

**Reviewers' Comments:**

Reviewer's Responses to Questions

**Key Review Criteria Required for Acceptance?**

As you describe the new analyses required for acceptance, please consider the following:

**Methods**

-Are the objectives of the study clearly articulated with a clear testable hypothesis stated?

-Is the study design appropriate to address the stated objectives?

-Is the population clearly described and appropriate for the hypothesis being tested?

-Is the sample size sufficient to ensure adequate power to address the hypothesis being tested?

-Were correct statistical analysis used to support conclusions?

-Are there concerns about ethical or regulatory requirements being met?

Reviewer #1: This duty enngbles some epidemiological data on lobo mycosis, an implantation mycosis. I have one observation related to the lack of professional data of the majority of the study population.

See attached file

Reviewer #2: The article entitled “Forest-river interfaces shape lobomycosis risk in the Amazon Basin” investigated effects of forest and water composition and configuration on lobomycosis occurrence while adjusting for relevant environmental and demographic covariates. Research is very important, especially since this is a disease about which very little is still known. However, to improve the study, the authors need to explore certain points in greater depth. The methodological analysis of the manuscript reveals a relevant, innovative, and overall well-conducted study, particularly for integrating neglected mycoses epidemiology with a quantitative geospatial approach. However, critical aspects require clarification or improvement to strengthen inferential robustness and reduce potential bias. First, the objectives are clearly articulated and aligned with a testable hypothesis, focusing on the influence of forest–river interfaces on lobomycosis occurrence. This represents an important conceptual contribution, as it shifts the traditional paradigm of exclusively forest-related exposure toward a more integrated landscape ecology framework. The coherence between the introduction, hypothesis, and statistical modeling is a clear strength.

The spatial case–control design is, in principle, appropriate for investigating environmental determinants. However, there are important limitations that should be more explicitly addressed. The main concern relates to the time span of the 192 cases. As described in the results (page 10), cases cover a wide temporal range (before 1979 to ≥2020), with concentration between 1980–1999. This temporal heterogeneity may introduce exposure misclassification bias, especially given substantial environmental changes in the Amazon (deforestation, hydrological alterations, urban expansion). Although the authors attempt to address this by estimating the “probable year of infection,” this variable is inherently imprecise and subject to recall bias and misclassification. Suggestion: perform sensitivity analyses stratified by time period (e.g., pre-2000 vs post-2000) or restrict analyses to a subset with higher temporal reliability. Alternatively, incorporate temporal uncertainty into the modeling framework (e.g., Bayesian approaches or time windows).

The study population is well described and epidemiologically consistent with the disease (male predominance, middle-aged adults, strong concentration in Acre). However, representativeness may be limited to cases identified in specific healthcare settings, raising concerns about selection bias. This is particularly relevant for neglected diseases with likely underreporting. The sample size (192 cases and 384 controls) is adequate for GAM-based analyses, especially considering nonlinear modeling. However, there is an important methodological ambiguity regarding the controls. Although described as “randomly selected within the same municipality” (page 7), it is not entirely clear whether they represent true absence of disease or simply absence of recorded cases. Moreover, there is no guarantee that these locations correspond to unexposed populations. Suggestion: clarify the epidemiological rationale for control selection (environmental pseudo-absences vs population-based controls). Ideally, discuss the possibility of differential misclassification bias and, if feasible, compare with independent data sources (e.g., areas with no historical records).

Another critical point concerns matching. While controls are “landscape-matched” at the municipal level, there is no individual matching, and municipalities in the Amazon can be highly heterogeneous. This may result in residual confounding. Suggestion: consider models with random effects at the municipality level or conditional analyses to better account for intra-municipality variability.

The georeferencing process is a methodological strength, combining secondary data, field validation, and GPS (page 7). Nevertheless, spatial error remains possible, particularly for older cases, potentially leading to attenuation bias in environmental associations. Suggestion: explicitly discuss the expected magnitude of spatial error and, if possible, conduct sensitivity analyses using larger buffers or spatial jittering.

The use of MapBiomas data and landscape metrics (PLAND and LPI) is appropriate and consistent with landscape epidemiology literature. The comparison between composition and configuration is a notable strength, and the finding that configuration (LPI) outperforms composition aligns with the ecological hypothesis. However, the analysis may be overly simplified by considering only two land cover classes (primary forest and water). Amazonian landscapes are highly heterogeneous, and elements such as secondary forest, floodplains, and anthropogenic land use may be relevant to pathogen ecology. Suggestion: incorporate additional metrics (e.g., edge density, fragmentation indices) or intermediate land cover classes (e.g., secondary forest, seasonally flooded areas) to better capture ecological interfaces.

The choice of buffer sizes (3 km² and 10 km²) is reasonable but lacks strong epidemiological justification. Although linked to human activity patterns (page 8) , this remains somewhat speculative. Suggestion: test additional spatial scales or apply a continuous multi-scale approach (e.g., moving window analysis) to strengthen ecological inference.

The statistical modeling using GAMs is appropriate and well implemented, including nonlinear relationships and spatial smooth terms. The absence of significant residual spatial autocorrelation (Moran’s I) is a strong point, indicating adequate control of spatial dependence. However, including both distance to rivers and water-related landscape metrics (LPI) may introduce collinearity, as both capture related environmental gradients. Suggestion: provide collinearity diagnostics (e.g., VIF or correlation matrix) and discuss potential redundancy among predictors. Interpretation of odds ratios also requires caution. For instance, the reported effect of water (LPI) as a “5-fold higher odds” per 10% increase appears exaggerated and may reflect scaling or nonlinear effects (page 13) . This should be clarified to avoid overinterpretation.

Regarding ethical aspects, although ethics approval and informed consent are reported (page 10), an important concern relates to retrospective geolocation data and the potential for indirect identification of vulnerable populations. While coordinate masking is mentioned, the method is not detailed. Suggestion: explicitly describe the spatial anonymization procedure and discuss residual re-identification risks in line with best practices in spatial epidemiology.

Finally, a major limitation is ecological inference: environmental associations are interpreted as proxies for individual risk without direct exposure data (e.g., behavior, trauma, water contact). Although acknowledged by the authors (page 16), this issue could be more critically discussed. Suggestion: emphasize the ecological nature of the findings and propose future studies incorporating individual-level exposure data (e.g., classical case–control or cohort designs).

In summary, this is a methodologically solid, innovative, and highly relevant study for lobomycosis epidemiology, with strong integration of environmental data and spatial modeling. Its main strengths include a clear hypothesis, advanced landscape metrics, robust statistical modeling, and significant conceptual contribution. The main limitations relate to temporal uncertainty, control definition, environmental simplification, and potential spatial and ecological biases. Addressing these issues would substantially enhance the study’s robustness and scientific impact.

Reviewer #3: The methods concerning the spatial analysis are robust and well explained.

In the introduction, please do not limit the spead of Lobomycosis to Brazil and Peru. Notably, please mention Jose A Suárez Emerg Infect Dis. 2023 regarding Panama, and Grotta et al. J Fungi 2023 (this reference is more recent and up to date than Sambourg et al)

Third paragraph of the introduction, regarding environmental routes and risks, please mention possible animal vectors and notably cite Gonçalves et al Emerg Infect Dis. 2025 (hypothesis of ticks as vectors)

In the methods, how did the authors manage the possible discrepancies between the patients' adresses and the presumed places of contamination? Did they always match? The incubation can be long and determining the place of contamination can be associated with bias

**********

**Results**

-Does the analysis presented match the analysis plan?

-Are the results clearly and completely presented?

-Are the figures (Tables, Images) of sufficient quality for clarity?

Reviewer #1: Please see the attached file.

Reviewer #2: The analytical component of this study is, overall, well aligned with the stated analysis plan, and this internal consistency represents a major strength. The use of generalized additive models (GAMs), as described in the Methods, is appropriately reflected in the Results, including the comparison between landscape composition (PLAND) and configuration (LPI), the evaluation across two spatial scales, and the use of model selection criteria (AIC). This coherence between planned and executed analyses enhances the credibility and reproducibility of the findings. Additionally, the inclusion of spatial smooth terms and the explicit assessment of residual spatial autocorrelation (Moran’s I) indicate a careful effort to address spatial dependence, which is often overlooked in similar studies.

The results are presented in a clear and structured manner, progressing logically from descriptive statistics to model selection and then to inferential outputs. The dataset description (page 10) provides an adequate epidemiological context, while Table 2 offers a transparent comparison of environmental variables between cases and controls. The subsequent model selection (Table 3) and effect estimates (Table 4) are well organized and facilitate interpretation of the main findings. The consistency of results across spatial scales further strengthens the robustness of the conclusions.

However, some aspects could be improved to enhance interpretability and avoid potential misinterpretation. First, while odds ratios (ORs) and confidence intervals are reported, the scaling of variables, particularly for landscape metrics such as LPI, is not always intuitive. For example, the interpretation of the effect of water bodies as a “5-fold increase” in odds may reflect nonlinear relationships or scaling choices rather than a directly interpretable epidemiological effect. Presenting marginal effects plots (as partially done in Figure 2) alongside clearer explanations of variable scaling would improve interpretability.

Second, although the GAM framework allows for nonlinear relationships, the manuscript could better emphasize how these nonlinearities influence the observed associations. The graphical outputs (Figure 2, page 14) are useful, but the text could more explicitly describe key thresholds or inflection points in the curves, which are often critical for ecological interpretation.

The visual elements of the manuscript are generally of high quality and contribute effectively to the understanding of the study. The study design figure (page 11) clearly illustrates the spatial framework, including case and control distribution, buffer construction, and environmental variables, which is particularly valuable for readers less familiar with spatial epidemiology. Tables are well formatted and contain sufficient detail to support the analysis. Figure 2 effectively conveys modeled relationships and uncertainty through confidence intervals.

That said, there are opportunities to further strengthen the visual presentation. The maps could include additional contextual layers, such as major river systems, administrative boundaries, or gradients of population density, to better situate the spatial patterns. Similarly, including density maps or kernel estimations of case distribution could help visualize clustering beyond point representation. For the statistical figures, adding rug plots or data density indicators would help readers assess the distribution of observations across the range of predictors.

Another point worth noting is that, while the results are clearly presented, there is limited exploration of potential interactions between variables (e.g., forest configuration × distance to river). Given the central hypothesis of ecological interfaces, explicitly testing interaction terms could provide stronger evidence for synergistic effects rather than independent associations.

Finally, although the analysis is robust and clearly presented, transparency could be further enhanced by briefly summarizing key modeling decisions (e.g., selection of smoothing parameters, handling of missing data, or model diagnostics) within the main text, rather than relying solely on methodological descriptions. This would improve accessibility for readers and facilitate reproducibility.

In summary, the analysis is coherent, well executed, and clearly presented, with high-quality visual elements that effectively support the findings. Minor improvements in the interpretation of effect sizes, exploration of nonlinearities and interactions, and enhancement of visual contextualization would further strengthen the analytical clarity and scientific impact of the study.

Reviewer #3: The results are well presented and clear;

**********

**Conclusions**

-Are the conclusions supported by the data presented?

-Are the limitations of analysis clearly described?

-Do the authors discuss how these data can be helpful to advance our understanding of the topic under study?

-Is public health relevance addressed?

Reviewer #1: Yes

Reviewer #2: The conclusions are consistent with the data presented and are appropriately supported by the analytical results, particularly the robust association between landscape configuration (LPI), proximity to rivers, and lobomycosis occurrence. The coherence between statistical findings and ecological interpretation strengthens the validity of the proposed role of forest–river interfaces as key environments for transmission .

The limitations are clearly acknowledged, especially regarding the use of environmental proxies and uncertainty in the timing of infection. This transparency is commendable and aligns with good practices in spatial epidemiology. The authors also adequately discuss how their findings contribute to a deeper understanding of lobomycosis, notably by reframing it as a disease influenced by ecological interfaces rather than solely by forest exposure.

The relevance to public health is well addressed, particularly in highlighting the importance of targeting riverine populations and incorporating environmental indicators into surveillance strategies. This is a significant contribution for neglected tropical diseases in remote Amazonian settings.

However, the discussion could be further strengthened by more explicitly integrating evidence from lobomycosis in aquatic animals, particularly dolphins, where similar lesions have been described. This body of evidence supports the hypothesis of an aquatic or semi-aquatic environmental reservoir and reinforces the biological plausibility of increased risk at forest–river interfaces. Incorporating this comparative perspective would enhance the ecological interpretation and provide a stronger interdisciplinary foundation.

Additionally, while the conclusions are well supported, they should be framed with caution to avoid overgeneralization at the individual level, given the ecological nature of the study. Emphasizing that the findings reflect population-level associations rather than direct exposure pathways would improve interpretative rigor.

Overall, the study provides meaningful and well-supported insights, with clear implications for advancing both scientific understanding and public health strategies related to lobomycosis.

Reviewer #3: The discussion could be partly rewritten

There are redundances, for example the paragraph "These findings suggest... riparina vegetation" is written twice.

How do we interpret these results, regarding possible water-associated hypotheses? the authors should give hints about possible future studies and research consequences regarding the different direct and indirect routes of infection (vector-borne, direct aquatic, etc)

**********

**Editorial and Data Presentation Modifications?**

Use this section for editorial suggestions as well as relatively minor modifications of existing data that would enhance clarity. If the only modifications needed are minor and/or editorial, you may wish to recommend “Minor Revision” or “Accept”.

Reviewer #1: Please use the term "implantation Mycosis" instead of "cutaneous mycosis"

Reviewer #2: Minor editorial and data presentation improvements could enhance clarity and reproducibility. First, explicitly define the temporal framework of case inclusion and clarify how the “probable year of infection” was operationalized. Second, better describe the selection and epidemiological meaning of control locations. Third, standardize the interpretation of odds ratios, particularly for scaled variables (e.g., LPI), to avoid overestimation of effects. In figures, include additional spatial context (e.g., main rivers, boundaries) and improve captions to clarify nonlinear relationships shown in GAM outputs. Finally, briefly report key model diagnostics (e.g., collinearity assessment, smoothing parameters) in the main text to improve transparency.

Reviewer #3: (No Response)

**********

**Summary and General Comments**

Use this section to provide overall comments, discuss strengths/weaknesses of the study, novelty, significance, general execution and scholarship. You may also include additional comments for the author, including concerns about dual publication, research ethics, or publication ethics. If requesting major revision, please articulate the new experiments that are needed.

Reviewer #1: Please see my attached file

Reviewer #2: This manuscript presents a relevant and innovative spatial epidemiological analysis of lobomycosis in the Amazon, with clear objectives, a coherent analytical framework, and appropriate use of geospatial data and generalized additive models. The study’s main strength lies in its conceptual contribution, shifting the paradigm from forest-only exposure to the role of forest–river interfaces, supported by consistent results across scales. The integration of landscape configuration metrics (LPI) and spatial modeling is methodologically robust and represents a significant advance in the field.

The results are clearly presented, supported by well-structured tables and informative figures, and the conclusions are generally consistent with the data. The discussion appropriately addresses public health relevance and acknowledges key limitations, particularly the use of environmental proxies.

However, several aspects require clarification or refinement. The wide temporal range of cases introduces potential exposure misclassification, and the estimation of infection timing should be better justified or explored through sensitivity analyses. The definition and epidemiological meaning of controls need clearer explanation, as they may represent pseudo-absences rather than true controls. Potential spatial and temporal biases (e.g., georeferencing uncertainty, landscape change over time) should be more explicitly discussed.

The environmental analysis could be strengthened by incorporating additional land cover classes or landscape metrics to better capture ecological complexity. Clarification of variable scaling and effect interpretation (e.g., odds ratios for LPI) is also needed to avoid over interpretation. Testing interactions between key variables (e.g., water–forest interface effects) would further support the central hypothesis.

Importantly, the discussion would benefit from integrating evidence of lobomycosis-like disease in aquatic animals (e.g., dolphins), reinforcing the plausibility of aquatic or semi-aquatic reservoirs.

No major ethical concerns are evident, although spatial anonymization methods should be more clearly described. Overall, the study is of high relevance and scientific merit, but would benefit from moderate revisions to improve clarity, methodological transparency, and ecological interpretation.

Reviewer #3: Interesting study on a very neglected disease

The main results are clearly presented and the spatial methods are solid

**********

PLOS authors have the option to publish the peer review history of their article (what does this mean?). If published, this will include your full peer review and any attached files.

If you choose “no”, your identity will remain anonymous but your review may still be made public.

**Do you want your identity to be public for this peer review?**  For information about this choice, including consent withdrawal, please see our Privacy Policy.

Reviewer #1: No

Reviewer #2: No

Reviewer #3: **Yes:**  Romain Blaizot

[NOTE: If reviewer comments were submitted as an attachment file, they will be attached to this email and accessible via the submission site. Please log into your account, locate the manuscript record, and check for the action link "View Attachments". If this link does not appear, there are no attachment files.]

**Figure resubmission:**

While revising your submission, we strongly recommend that you use PLOS’s NAAS tool (https://ngplosjournals.pagemajik.ai/artanalysis) to test your figure files. NAAS can convert your figure files to the TIFF file type and meet basic requirements (such as print size, resolution), or provide you with a report on issues that do not meet our requirements and that NAAS cannot fix.

After uploading your figures to PLOS’s NAAS tool - https://ngplosjournals.pagemajik.ai/artanalysis, NAAS will process the files provided and display the results in the "Uploaded Files" section of the page as the processing is complete. If the uploaded figures meet our requirements (or NAAS is able to fix the files to meet our requirements), the figure will be marked as "fixed" above. If NAAS is unable to fix the files, a red "failed" label will appear above. When NAAS has confirmed that the figure files meet our requirements, please download the file via the download option, and include these NAAS processed figure files when submitting your revised manuscript.

**Reproducibility:**

To enhance the reproducibility of your results, we recommend that authors of applicable studies deposit laboratory protocols in protocols.io, where a protocol can be assigned its own identifier (DOI) such that it can be cited independently in the future. Additionally, PLOS ONE offers an option to publish peer-reviewed clinical study protocols. Read more information on sharing protocols at https://plos.org/protocols?utm_medium=editorial-email&utm_source=authorletters&utm_campaign=protocols

Attachment Submitted filename: Lobomycosis Comments.docx

# Author comment: Author response to Decision Letter 1

**DOI:** 10.1371/journal.pntd.0014502.r002

14 May 2026

Attachment Submitted filename: Response_to_Reviewers_signed.pdf

# Peer review: Decision Letter 1

**DOI:** 10.1371/journal.pntd.0014502.r003

**Authors:** Marcio L Rodrigues, David Simons

10 Jun 2026

PNTD-D-26-00616R1

Forest-river interfaces shape lobomycosis risk in the Amazon Basin

PLOS Neglected Tropical Diseases

Dear Dr. Laporta,

Thank you for submitting your manuscript to PLOS Neglected Tropical Diseases. After careful consideration, we feel that it has merit but does not fully meet PLOS Neglected Tropical Diseases's publication criteria as it currently stands. Therefore, we invite you to submit a revised version of the manuscript that addresses the points raised during the review process.

Please submit your revised manuscript by Aug 09 2026 11:59PM. If you will need more time than this to complete your revisions, please reply to this message or contact the journal office at plosntds@plos.org. When you're ready to submit your revision, log on to https://www.editorialmanager.com/pntd/ and select the 'Submissions Needing Revision' folder to locate your manuscript file.

Please include the following items when submitting your revised manuscript:

* A letter that responds to each point raised by the editor and reviewer(s). You should upload this letter as a separate file labeled 'Response to Reviewers'. This file does not need to include responses to any formatting updates and technical items listed in the 'Journal Requirements' section below.

* A marked-up copy of your manuscript that highlights changes made to the original version. You should upload this as a separate file labeled 'Revised Manuscript with Track Changes'.

* An unmarked version of your revised paper without tracked changes. You should upload this as a separate file labeled 'Manuscript'.

If you would like to make changes to your financial disclosure, competing interests statement, or data availability statement, please make these updates within the submission form at the time of resubmission. Guidelines for resubmitting your figure files are available below the reviewer comments at the end of this letter.

As the corresponding author, your ORCID iD is verified in the submission system and will appear in the published article. PLOS supports the use of ORCID, and we encourage all coauthors to register for an ORCID iD and use it as well. Please encourage your coauthors to verify their ORCID iD within the submission system before final acceptance, as unverified ORCID iDs will not appear in the published article. Only the individual author can complete the verification step; PLOS staff cannot verify ORCID iDs on behalf of authors.

We look forward to receiving your revised manuscript.

Kind regards,

David Simons

Academic Editor

PLOS Neglected Tropical Diseases

Marcio Rodrigues

Section Editor

PLOS Neglected Tropical Diseases

Shaden Kamhawi

co-Editor-in-Chief

PLOS Neglected Tropical Diseases

orcid.org/0000-0003-4304-636XX

Paul Brindley

co-Editor-in-Chief

PLOS Neglected Tropical Diseases

orcid.org/0000-0003-1765-0002

**Additional Editor Comments (if provided):**

The reviewers and I appreciate the thoroughness with which you addressed the concerns raised in the previous round. The integration of landscape ecology concepts with spatial epidemiology is robust, and your revisions have strengthened the manuscript.

We are happy to recommended publication subject to a few final, minor revisions. Because these are straightforward, I will evaluate your next revision editorially without sending the manuscript back out for external peer review.

Please carefully review the odds ratios reported in Table 4 alongside your textual interpretation of these values in the Results section. There appear to be potential inconsistencies between the statistical output and the narrative description of the effect sizes. Please ensure the scaling and magnitude of these effects are described accurately and without overestimation.

While you have adequately addressed the temporal uncertainty of the retrospective design, please explicitly add the absence of a formal power analysis/sample size calculation as a methodological limitation in the Discussion section.

Kind regards,

David

**Journal Requirements:**

If the reviewer comments include a recommendation to cite specific previously published works, please review and evaluate these publications to determine whether they are relevant and should be cited. There is no requirement to cite these works unless the editor has indicated otherwise.

1) Thank you for stating the Data availability " To support peer review and reproducibility, an anonymized 10-km2 analytical dataset with spatially jittered coordinates and the full R analysis script have been provided as review files for editors and reviewers. Original geographic coordinates are not shared to protect participant confidentiality. **Upon acceptance, the anonymized datasets, spatially jittered coordinates required to reproduce the spatial smooth terms, derived environmental variables** , and the full native R script will be deposited in the Zenodo repository (doi:10.5281/zenodo.19287860)."

We strongly recommend all authors deposit their data before acceptance, as the process can be lengthy and hold up publication timelines. Please note that, though access restrictions are acceptable now, your entire minimal dataset will need to be made freely accessible if your manuscript is accepted for publication. This policy applies to all data except where public deposition would breach compliance with the protocol approved by your research ethics board. If you are unable to adhere to our open data policy, please kindly revise your statement to explain your reasoning and we will seek the editor's input on an exemption.

**Reviewers' comments:**

Reviewer's Responses to Questions

**Key Review Criteria Required for Acceptance?**

As you describe the new analyses required for acceptance, please consider the following:

**Methods**

-Are the objectives of the study clearly articulated with a clear testable hypothesis stated?

-Is the study design appropriate to address the stated objectives?

-Is the population clearly described and appropriate for the hypothesis being tested?

-Is the sample size sufficient to ensure adequate power to address the hypothesis being tested?

-Were correct statistical analysis used to support conclusions?

-Are there concerns about ethical or regulatory requirements being met?

Reviewer #2: The study population is well described, including demographic, occupational, temporal, and geographic details. It is representative of the known epidemiology of lobomycosis in the Amazon Basin. The dataset includes 192 georeferenced cases and 384 controls. This is substantial for such a rare disease and likely sufficient for the analyses, although no formal sample size or power calculation was included.

The statistical approach is robust and appropriate. The use of generalized additive models (GAMs), adjustment for relevant environmental covariates, model comparison using AICc, and assessment of residual spatial autocorrelation through Moran’s I provides confidence in the analytical framework. Ethical requirements appear to have been fully met, including institutional review board approval, informed consent procedures, and adequate measures to protect participant confidentiality. The availability of data and code in a public repository further strengthens transparency and reproducibility.

Overall, the Methods section is scientifically sound and substantially improved compared with earlier versions. The main remaining limitation is the uncertainty associated with the estimated year of infection, which may introduce temporal mismatches between environmental conditions and disease acquisition. However, the authors acknowledge this issue, and it does not substantially undermine the study's conclusions.

**********

**Results**

-Does the analysis presented match the analysis plan?

-Are the results clearly and completely presented?

-Are the figures (Tables, Images) of sufficient quality for clarity?

Reviewer #2: The analyses presented are consistent with the methods and analytical plan described in the manuscript. The authors clearly report descriptive statistics, model selection procedures, and the results of the final GAM models. The comparison between landscape composition and configuration metrics is well structured and supports the study’s central hypothesis.

Results are presented clearly and logically. The tables effectively summarize demographic characteristics, environmental variables, model selection outcomes, and effect estimates. The figures are generally informative and facilitate understanding of both the study design and the modeled relationships between environmental variables and disease occurrence.

Minor improvements could still be made. The interpretation of odds ratios, particularly for the Largest Patch Index (LPI), would benefit from clarification, as the magnitude of some reported effects appears inconsistent with the scale of the variables. Additionally, Figure 2 could be improved by harmonizing axis scales and enhancing visual clarity for publication.

Despite these minor issues, the Results section is complete, transparent, and adequately supports the main findings.

**********

**Conclusions**

-Are the conclusions supported by the data presented?

-Are the limitations of analysis clearly described?

-Do the authors discuss how these data can be helpful to advance our understanding of the topic under study?

-Is public health relevance addressed?

Reviewer #2: The conclusions are supported by the data presented and remain consistent with the statistical analyses. The study provides compelling evidence that landscape configuration at forest–river interfaces is more strongly associated with lobomycosis occurrence than forest cover alone, representing an important conceptual advance in understanding the environmental ecology of this neglected mycosis.

The study's limitations are appropriately acknowledged, particularly its reliance on environmental proxies and the uncertainty surrounding the probable year of infection. Although additional discussion of potential spatial misclassification and selection bias could further strengthen the manuscript, the authors adequately recognize the main sources of uncertainty.

Importantly, the manuscript advances current knowledge by proposing a refined ecological framework for lobomycosis transmission. The findings suggest that forest–river interfaces may serve as ecological hotspots where environmental suitability and human exposure converge. The discussion would be further strengthened by integrating evidence on lobomycosis-like disease in aquatic mammals, particularly dolphins, which could provide additional biological plausibility for the observed associations with aquatic environments.

The public health relevance is clearly articulated. The study identifies environmental indicators that may support surveillance and prevention strategies among remote Amazonian populations and provides valuable information to target high-risk areas.

**********

**Editorial and Data Presentation Modifications?**

Use this section for editorial suggestions as well as relatively minor modifications of existing data that would enhance clarity. If the only modifications needed are minor and/or editorial, you may wish to recommend “Minor Revision” or “Accept”.

Reviewer #2: Only minor revisions are recommended. The manuscript would benefit from a clearer explanation of the scaling used to calculate odds ratios, particularly for landscape metrics. A brief justification for the absence of a formal power calculation would also be useful. In addition, the discussion could more explicitly address potential biases associated with historical georeferencing and temporal uncertainty.

Figure 2 could be visually improved for publication, and minor formatting adjustments in tables and figure legends should be considered. These issues are editorial in nature and do not affect the validity of the results or conclusions.

**********

**Summary and General Comments**

Use this section to provide overall comments, discuss strengths/weaknesses of the study, novelty, significance, general execution and scholarship. You may also include additional comments for the author, including concerns about dual publication, research ethics, or publication ethics. If requesting major revision, please articulate the new experiments that are needed.

Reviewer #2: This manuscript presents a novel and methodologically sound spatial epidemiological investigation of lobomycosis in the Amazon Basin. The study addresses an important gap in the understanding of the environmental determinants of one of the most neglected fungal diseases worldwide. The integration of landscape ecology concepts with spatial epidemiology represents a significant strength and provides valuable insights into disease transmission dynamics.

The authors have satisfactorily addressed the major concerns raised during review, including clarification of the hypothesis, study design, control selection, ethical considerations, and data availability. The analytical framework is robust, and the conclusions are generally well supported by the evidence presented.

The principal strengths of the study include its originality, relatively large sample size for a rare disease, use of geospatial methods, transparent analytical approach, and clear public health relevance. Remaining limitations are primarily related to temporal uncertainty in estimating infection dates and the inherent constraints of retrospective environmental analyses.

Overall, this work represents a meaningful contribution to the fields of medical mycology, spatial epidemiology, and neglected tropical diseases. Subject to minor revisions, I believe the manuscript is suitable for publication.

**********

PLOS authors have the option to publish the peer review history of their article (what does this mean?). If published, this will include your full peer review and any attached files.

If you choose “no”, your identity will remain anonymous but your review may still be made public.

**Do you want your identity to be public for this peer review?**  For information about this choice, including consent withdrawal, please see our Privacy Policy.

Reviewer #2: No

[NOTE: If reviewer comments were submitted as an attachment file, they will be attached to this email and accessible via the submission site. Please log into your account, locate the manuscript record, and check for the action link "View Attachments". If this link does not appear, there are no attachment files.]

**Figure resubmission:**

While revising your submission, we strongly recommend that you use PLOS’s NAAS tool (https://ngplosjournals.pagemajik.ai/artanalysis) to test your figure files. NAAS can convert your figure files to the TIFF file type and meet basic requirements (such as print size, resolution), or provide you with a report on issues that do not meet our requirements and that NAAS cannot fix.

After uploading your figures to PLOS’s NAAS tool - https://ngplosjournals.pagemajik.ai/artanalysis, NAAS will process the files provided and display the results in the "Uploaded Files" section of the page as the processing is complete. If the uploaded figures meet our requirements (or NAAS is able to fix the files to meet our requirements), the figure will be marked as "fixed" above. If NAAS is unable to fix the files, a red "failed" label will appear above. When NAAS has confirmed that the figure files meet our requirements, please download the file via the download option, and include these NAAS processed figure files when submitting your revised manuscript.

**Reproducibility:**

To enhance the reproducibility of your results, we recommend that authors of applicable studies deposit laboratory protocols in protocols.io, where a protocol can be assigned its own identifier (DOI) such that it can be cited independently in the future. Additionally, PLOS ONE offers an option to publish peer-reviewed clinical study protocols. Read more information on sharing protocols at https://plos.org/protocols?utm_medium=editorial-email&utm_source=authorletters&utm_campaign=protocols

# Author comment: Author response to Decision Letter 2

**DOI:** 10.1371/journal.pntd.0014502.r004

19 Jun 2026

Attachment Submitted filename: Response_to_Reviewers_signed_auresp_2.pdf

# Editor report: Decision Letter 2

**DOI:** 10.1371/journal.pntd.0014502.r005

**Authors:** Marcio L Rodrigues, David Simons

24 Jun 2026

Dear Dr. Laporta,

We are pleased to inform you that your manuscript 'Forest-river interfaces shape lobomycosis risk in the Amazon Basin' has been provisionally accepted for publication in PLOS Neglected Tropical Diseases.

Before your manuscript can be formally accepted you will need to complete some formatting changes, which you will receive in a follow up email. A member of our team will be in touch with a set of requests.

Please note that your manuscript will not be scheduled for publication until you have made the required changes, so a swift response is appreciated.

IMPORTANT: The editorial review process is now complete. PLOS will only permit corrections to spelling, formatting or significant scientific errors from this point onwards. Requests for major changes, or any which affect the scientific understanding of your work, will cause delays to the publication date of your manuscript.

Should you, your institution's press office or the journal office choose to press release your paper, you will automatically be opted out of early publication. We ask that you notify us now if you or your institution is planning to press release the article. All press must be co-ordinated with PLOS.

Thank you again for supporting Open Access publishing; we are looking forward to publishing your work in PLOS Neglected Tropical Diseases.

Best regards,

Marcio Rodrigues

Section Editor

PLOS Neglected Tropical Diseases

Shaden Kamhawi

co-Editor-in-Chief

PLOS Neglected Tropical Diseases

orcid.org/0000-0003-4304-636XX

Paul Brindley

co-Editor-in-Chief

PLOS Neglected Tropical Diseases

orcid.org/0000-0003-1765-0002

***********************************************************

p.p1 {margin: 0.0px 0.0px 0.0px 0.0px; line-height: 16.0px; font: 14.0px Arial; color: #323333; -webkit-text-stroke: #323333}span.s1 {font-kerning: none

# Editor report: Acceptance letter

**DOI:** 10.1371/journal.pntd.0014502.r006

**Authors:** Marcio L Rodrigues, David Simons

Dear Dr. Laporta,

We are delighted to inform you that your manuscript, "

Forest-river interfaces shape lobomycosis risk in the Amazon Basin," has been formally accepted for publication in PLOS Neglected Tropical Diseases.

We have now passed your article onto the PLOS Production Department who will complete the rest of the publication process. All authors will receive a confirmation email upon publication.

The corresponding author will soon be receiving a typeset proof for review, to ensure errors have not been introduced during production. Please review the PDF proof of your manuscript carefully, as this is the last chance to correct any scientific or type-setting errors. Please note that major changes, or those which affect the scientific understanding of the work, will likely cause delays to the publication date of your manuscript. Note: Proofs for Front Matter articles (Editorial, Viewpoint, Symposium, Review, etc...) are generated on a different schedule and may not be made available as quickly.

Soon after your final files are uploaded, the early version of your manuscript will be published online unless you opted out of this process. The date of the early version will be your article's publication date. The final article will be published to the same URL, and all versions of the paper will be accessible to readers.

For Research Articles, you will receive an invoice from PLOS for your publication fee after your manuscript has reached the completed accept phase. If you receive an email requesting payment before acceptance or for any other service, this may be a phishing scheme. Learn how to identify phishing emails and protect your accounts at https://explore.plos.org/phishing.

Thank you again for supporting open-access publishing; we are looking forward to publishing your work in PLOS Neglected Tropical Diseases.

Best regards,

Shaden Kamhawi

co-Editor-in-Chief

PLOS Neglected Tropical Diseases

Paul Brindley

co-Editor-in-Chief

PLOS Neglected Tropical Diseases
